# Wilms’ tumor 1 (WT1) antigen is overexpressed in Kaposi Sarcoma and is regulated by KSHV vFLIP

**DOI:** 10.1371/journal.ppat.1011881

**Published:** 2024-01-08

**Authors:** Ayana E. Morales, Ruby Gumenick, Caitlyn M. Genovese, Yun Yeong Jang, Ariene Ouedraogo, Maite Ibáñez de Garayo, Tania Pannellini, Sanjay Patel, Matthew E. Bott, Julio Alvarez, Sung Soo Mun, Jennifer Totonchy, Archana Gautam, Jesus Delgado de la Mora, Stephanie Chang, Dagmar Wirth, Marcelo Horenstein, Tao Dao, David A. Scheinberg, Paul G. Rubinstein, Aggrey Semeere, Jeffrey Martin, Catherine C. Godfrey, Carlee B. Moser, Roy M. Matining, Thomas B. Campbell, Margaret Z. Borok, Susan E. Krown, Ethel Cesarman

**Affiliations:** 1 Department of Medicine, Weill Cornell Medicine, New York, New York, United States of America; 2 Department of Pathology and Laboratory Medicine, Weill Cornell Medicine, New York, New York, United States of America; 3 Molecular Pharmacology Program, Memorial Sloan Kettering Cancer Center, New York, New York, United States of America; 4 School of Pharmacy, Chapman University, Irvine, California, United States of America; 5 Department of Allergy and Immunology, Icahn School of Medicine, New York, New York, United States of America; 6 Cornell University, Ithaca, New York, United States of America; 7 Model Systems for Infection and Immunity, Helmholtz Centre for Infection Research Braunschweig, Germany; 8 Section of Hematology/Oncology, John H. Stroger Jr Hospital of Cook County (Cook County Hospital), Ruth M. Rothstein Core Center, University of Illinois, Chicago, Illinois, United States of America; 9 Infectious Diseases Institute, College of Health Sciences, Makerere University, Kampala, Uganda; 10 Department of Epidemiology and Biostatistics, University of California, San Francisco, California, United States of America; 11 Office of the Global AIDS Coordinator, Department of State, Washington, DC, United States of America; 12 Center for Biostatistics in AIDS Research, Harvard T H Chan School of Public Health, Boston, Massachusetts, United States of America; 13 Department of Medicine, University of Colorado School of Medicine, Aurora, Colorado, United States of America; 14 Department of Internal Medicine, University of Zimbabwe, Harare, Zimbabwe; 15 Memorial Sloan Kettering Cancer Center (emerita), New York, New York, United States of America; University of North Carolina at Chapel Hill, UNITED STATES

## Abstract

In people living with HIV, Kaposi Sarcoma (KS), a vascular neoplasm caused by KS herpesvirus (KSHV/HHV-8), remains one of the most common malignancies worldwide. Individuals living with HIV, receiving otherwise effective antiretroviral therapy, may present with extensive disease requiring chemotherapy. Hence, new therapeutic approaches are needed. The Wilms’ tumor 1 (WT1) protein is overexpressed and associated with poor prognosis in several hematologic and solid malignancies and has shown promise as an immunotherapeutic target. We found that WT1 was overexpressed in >90% of a total 333 KS biopsies, as determined by immunohistochemistry and image analysis. Our largest cohort from ACTG, consisting of 294 cases was further analyzed demonstrating higher WT1 expression was associated with more advanced histopathologic subtypes. There was a positive correlation between the proportion of infected cells within KS tissues, assessed by expression of the KSHV-encoded latency-associated nuclear antigen (LANA), and WT1 positivity. Areas with high WT1 expression showed sparse T-cell infiltrates, consistent with an immune evasive tumor microenvironment. We show that major oncogenic isoforms of WT1 are overexpressed in primary KS tissue and observed WT1 upregulation upon de novo infection of endothelial cells with KSHV. KSHV latent viral FLICE-inhibitory protein (vFLIP) upregulated total and major isoforms of WT1, but upregulation was not seen after expression of mutant vFLIP that is unable to bind IKKƴ and induce NFκB. siRNA targeting of WT1 in latent KSHV infection resulted in decreased total cell number and pAKT, BCL2 and LANA protein expression. Finally, we show that ESK-1, a T cell receptor–like monoclonal antibody that recognizes WT1 peptides presented on MHC HLA-A0201, demonstrates increased binding to endothelial cells after KSHV infection or induction of vFLIP expression. We propose that oncogenic isoforms of WT1 are upregulated by KSHV to promote tumorigenesis and immunotherapy directed against WT1 may be an approach for KS treatment.

## Author summary

Kaposi sarcoma (KS) is a vascular neoplasm caused by the Kaposi sarcoma herpesvirus (KSHV/HHV-8). People living with HIV have a significantly higher risk of developing KS and often have a more aggressive clinical course. Although effective antiretroviral therapy may lead to regression of HIV-associated KS lesions, advanced cases of KS also require chemotherapy, which is rarely curative. Wilms’ tumor 1 (WT1) has been reported to be highly expressed in various cancers, functioning as an oncogene and associated with a poor prognosis. WT1 is also an important immunotherapeutic target, with several WT1-directed therapies shown to be safe and demonstrating antineoplastic activity in early clinical trials for leukemias and solid tumors. We report high expression of WT1 in KS. We found that latent KSHV infection in endothelial cells leads to WT1 upregulation and that this effect is at least partly mediated by vFLIP, a protein produced by KSHV during latent viral infection. This study advances our understanding of how KSHV and a host cellular protein, WT1, interact, possibly to promote tumorigenesis and importantly, to support the pre-clinical rationale for conducting anti-WT1 immunotherapy trials in KS.

## Introduction

Kaposi sarcoma herpesvirus (KSHV), also called human herpesvirus 8 (HHV-8), the etiologic agent of Kaposi sarcoma (KS), has been classified as a carcinogen by the International Agency for Research on Cancer (IARC) [1]. KSHV viral DNA and protein are detected in all KS tumors [2,3]. KS is a vascular neoplasm that may present as a few lesions confined to the skin, but may progress to multiple tumors that can involve the oral mucosa, lymphatics, lungs and other visceral organs [3]. Morphologically, KS lesions may present as patches, plaques and nodules, most frequently on the lower extremities [4]. Despite effective antiretroviral therapy (ART), KS remains one of the most common cancers in people living with human immunodeficiency virus (HIV) globally [5,6]. While KS also occurs in people without HIV infection, HIV/AIDS-associated KS is generally the most aggressive form, occurring most often in HIV and KSHV-co-infected individuals in sub-Saharan Africa and elsewhere in men who have sex with men (MSM) [5–9]. People living with HIV (PLWH) may have an up to a 500-fold excess relative risk for the development of KS compared to the general population [8–10]. Furthermore, KS continues to occur in PLWH despite HIV suppression with antiretroviral therapy [10]. While ART alone may sometimes lead to tumor regression, chemotherapy is usually required for patients with advanced KS, is rarely curative, and incurs a risk for severe toxicities [7,12–15]. While immunosuppression plays a role in KS progression, additional viral and host factors are involved. Further studies of the molecular and cellular mechanisms involved in the pathobiology of KS may help uncover potential targets of therapy as well as prognostic biomarkers.

Wilms’ Tumor 1 (WT1), located on chromosome 11p13, is a C2H2 zinc-finger transcription factor that was initially discovered as a tumor suppressor [16]. However, when overexpressed, it promotes carcinogenesis in various hematological malignancies and solid tumors [17,18]. WT1 has significant roles in embryogenesis and constitutive WT1 knockout results in embryonic lethality [19–23]. Wild type WT1 is expressed sparsely in isolated sites in adult tissues, but is frequently aberrantly overexpressed in human leukemias and solid tumors [16–18,24,25]. High expression of WT1 is associated with poor prognosis in AML, myelodysplastic syndromes and several solid tumors [17,18]. There are at least 36 different WT1 isoforms resulting from alternative transcription initiation sites, alternative splice variants, different translation initiation sites, mRNA editing, and posttranslational modifications [24,26]. Alternative splicing accounts for the four major WT1 isoforms (A-D) which differ by the presence or lack of the 17AA insert (17 amino acids encoded by exon 5) and the KTS insert (the three amino acids lysine, threonine, serine), encoded by the end sequence of exon 9 [16]. In addition, the functions of WT1 are known to differ based on the cellular context [27]. These four major WT1 isoforms are overexpressed in solid tumors and leukemias, having varying oncogenic functions that promote cellular proliferation [26], survival [26,28–30], angiogenesis [31], cell migration and invasion [32], anti-apoptotic actions [33], and contribute to epithelial to mesenchymal transition [34].

In 2009, the National Cancer Institute (NCI) ranked WT1 as the number one cancer antigen toward which to direct immunotherapy research, based on overall criteria assessing therapeutic function, immunogenicity, oncogenicity, specificity, expression level, stem cell expression, number of antigen positive cancers, antigenic epitopes, and cellular location of antigen expression [35]. Immunotherapy targeted against WT1 in various hematological malignancies and solid cancers has induced immunological and clinical responses in preclinical studies [36] and clinical trials for solid tumors and hematologic malignancies [37–39]. Immunotherapeutic approaches directed against WT1 include T cell receptor mimic monoclonal antibodies, peptide vaccines (now entering Phase 3 clinical trials), and peptide pulsed or mRNA loaded dendritic cell vaccines, as well cytotoxic T cell lymphocytes (CTLs) [36–42]. These trials so far have shown safety as well as clinical and immunological responses. Moreover, WT1 vaccination is being studied in combination with immune checkpoint inhibitors in patients with relapsed or refractory solid tumors and leukemias (ClinicalTrials.gov, NCT03761914). Recently, donor-derived, EBV-specific CD8+T cells with high affinity WT1 specific TCR (TCR_C4_) generated persistent T cell responses in 12 patients with high risk AML post allogeneic hematopoietic cell transplantation (HCT), with higher relapse-free survival than HCT alone [43].

Like other herpesviruses, KSHV can establish lifelong latency, and in KSHV-induced tumors, the majority of cells are latently infected [44,45]. KSHV expresses a latency locus, consisting of LANA, vCyclin, vFLIP, microRNAs and Kaposin, which are thought to drive cellular transformation [46,47]. In particular, the latent gene viral FLICE inhibitory protein (vFLIP) induces vascular proliferation, spindle cell morphology and an inflammatory phenotype when expressed in endothelial cells [48]. vFLIP activates IκB kinase 1 (IKK1) to stimulate nuclear factor κB (NFκB) signaling to increase cell survival by directly binding to IKKƴ (NEMO) [48,49]. There are putative NFκB binding sites within the WT1 promoter, and ectopic expression of p50 and p65 subunits of NFκB upregulate WT1 promoter activity [50]. An analysis of vFLIP ectopic expression on human umbilical vein endothelial cells (HUVEC) followed by cDNA microarray analysis, showed that WT1 RNA was among a number of upregulated transcripts [51].

WT1 expression was previously examined in a variety of vascular tumors, including a few cases of non-HIV-associated KS, and expression was reported in 8/19 cases [52,53]. However, the spectrum of WT1 expression in KS is not well studied, and neither the mechanism of WT1 upregulation nor the spatial relationship of WT1 and immune infiltrates in KS have been previously investigated. Hence, we evaluated baseline WT1 expression in KS tumors in a large group of individuals with advanced HIV-associated KS who participated in a prospectively randomized therapeutic trial [54], plus additional cohorts of both non-HIV and HIV-associated KS. We also investigated whether KSHV plays a direct role in WT1 expression and investigated the effects of decreased WT1 in latent *in vitro* KSHV infection.

## Results

### WT1 is overexpressed in KS and correlates with LANA expression

**Fig 1:**
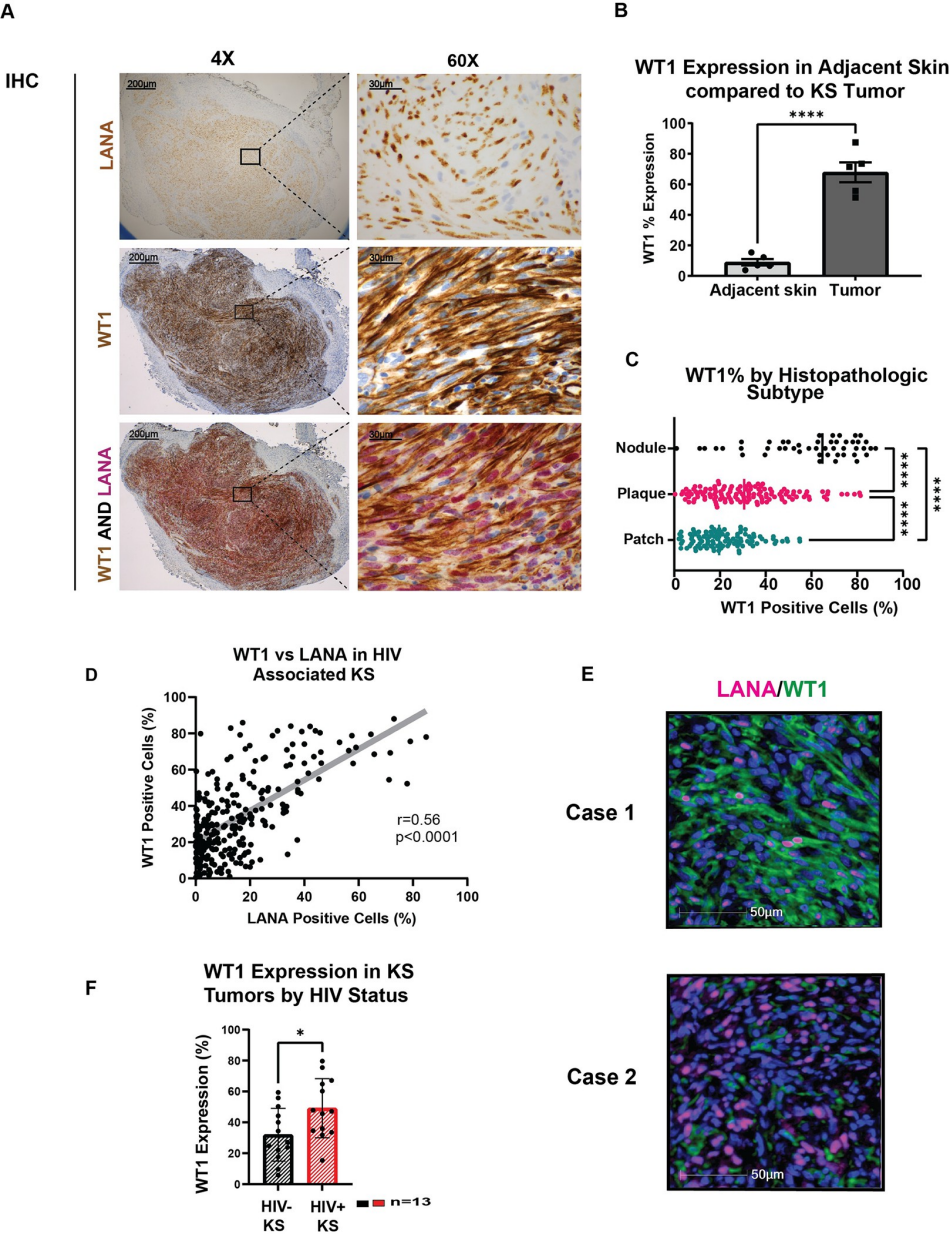
WT1 is Overexpressed in Kaposi Sarcoma. **A.** A representative KS case showing KSHV LANA (brown, nuclear) (top panel, with zoom-in on right panels), WT1 (brown, nuclear and cytoplasmic) (middle panels), and by double IHC for WT1 (brown, nuclear and cytoplasmic and KSHV LANA (red, nuclear) (lower panels) (Original magnification, 20X). **B.** Five KS tissue biopsies were analyzed using HALO analysis software for WT1 in the lesional tissue and adjacent normal subcutaneous tissue, demonstrating significant differential WT1 expression in KS compared to normal skin, p< 0.0001(***) using a two-sided unpaired t-test. **C.** Higher proportion of WT1-positive cells occurred with advanced histopathologic subtype demonstrated by scatter plot, patch vs. plaque p<0.0001(****), patch vs. nodule, p<0.0001(****), and plaque vs. nodule, p<0.0001(****), using Tukey’s multiple comparison test. WT1 analysis according to histopathologic subtype is also demonstrated in **S1 Table. D.** Proportion of WT1 expressing cells correlated significantly with proportion of LANA+ cells, r = 0.5564, p<0.0001(****), as determined by IHC and image analysis using Spearman’s correlation analysis, N = 261. **E**. The VECTRA panels performed of WT1/LANA demonstrating representative examples in Case 1 and Case 2 of the heterogeneity of KS tumors, with WT1 at times colocalizing (Case 1) and other times in neighboring but separate cells (Case 2). **F**. WT1 expression examined in 13 tumor biopsies from HIV positive and 13 from HIV negative patients, demonstrating increased WT1 expression in the HIV positive group, p = .023(*) using a two-sided unpaired t-test.

KS tumor biopsies from 294 PLWH and advanced stage AIDS-Kaposi sarcoma in a multinational clinical trial (ACTG A5263/AMC 066; NCT01435018) [54] were sectioned for H&E, and immunohistochemistry (IHC) for WT1 and LANA was performed (**Fig 1A**). Double IHC for both WT1 and LANA revealed WT1 expression in many LANA+ cells (**Fig 1A**). WT1 was overexpressed in areas involved by KS, but not in adjacent normal skin within the same biopsy (p< 0.0001; **Fig 1B**) with over 89% of cases demonstrating overexpression, i.e., higher expression than the mean expression found in adjacent normal skin (**Fig 1B**). LANA expression was nuclear as expected, and WT1 expression was found both in the nuclei and cytoplasm as previously noted in studies of solid tumors [55], although it was predominantly cytoplasmic in KS tissues (**Fig 1A**). Histopathologic classification was performed as previously described [4]. WT1 expression correlated with advanced histopathologic subtype, with highest expression in nodule subtypes (p<0.0001; **Fig 1C**). Furthermore, WT1 expression was positively correlated with expression of LANA (r = 0.56, p<0.0001; **Fig 1D**). LANA expression was also significantly associated with the histopathologic subtype, with high expression among nodule subtypes (p <0.001; **S1 Table**). Multiparameter imaging was performed as previously described [56] of WT1 and LANA confirmed that WT1 and LANA were in the same areas, at times co-localizing within the same cell or in neighboring but separate cells (**Fig 1E** top and bottom panels, respectively).

Additional validation cohorts were examined by IHC (**S2A–S2E Tables**). Among cases obtained in the US, a paired cohort with a range of histologies that were similar in number (patch, plaques, and nodules) from 13 HIV-positive and 13 HIV-negative patients showed significantly higher WT1 expression in the HIV-positive cohort (p = 0.023) (**Fig 1F and S2D and S2E Tables**). The patient characteristics of this cohort of PLWH (n = 13) and a cohort of HIV negative (n = 13) are compared in **S3 Table**.

**Fig 2:**
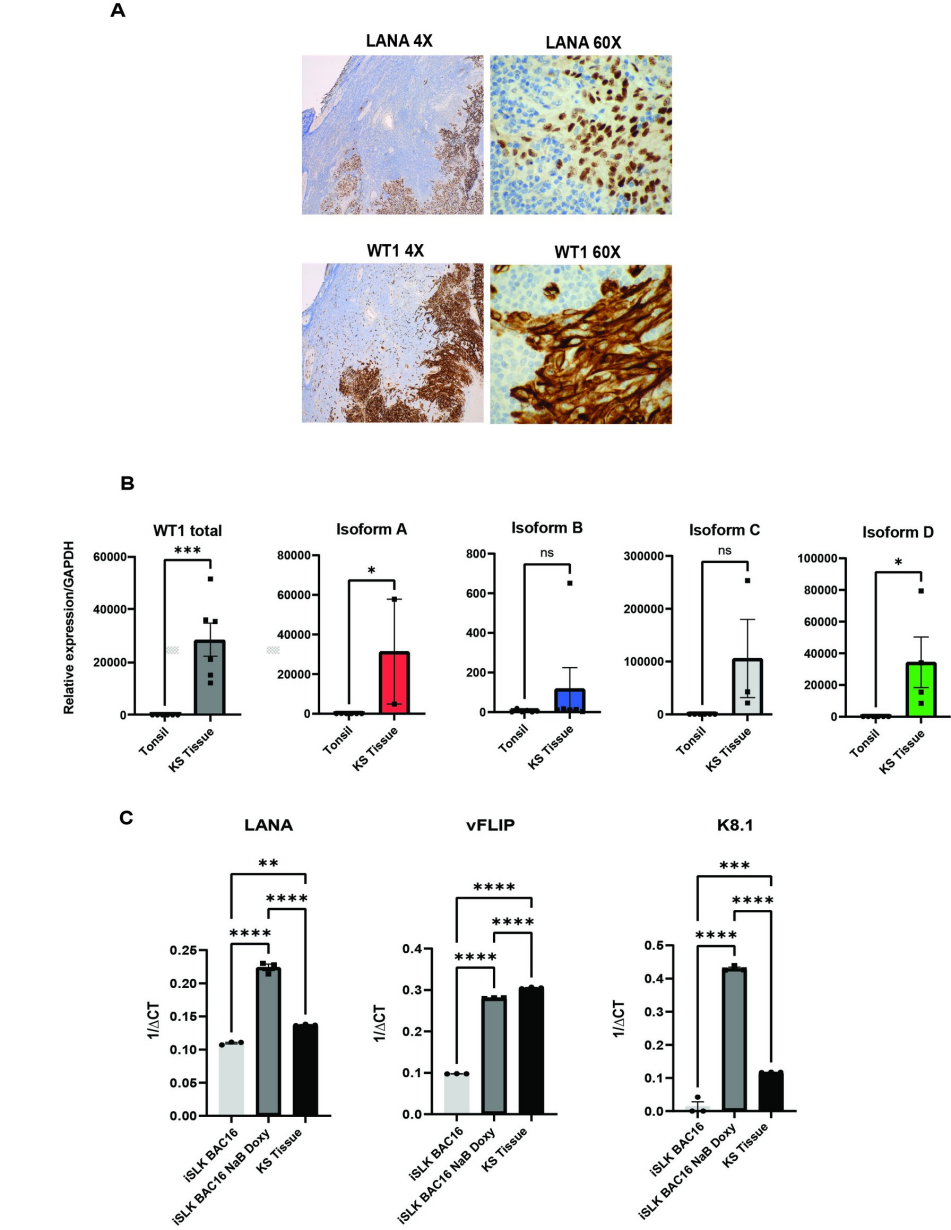
Major Oncogenic WT1 Isoforms are expressed in Primary KS tissue. **A.** Representative immunohistochemistry of KS within a lymph node used for RNA extraction to evaluate WT1 isoform expression. **B.** RT-qPCR of total WT1, p = 0.0010 (***) and major WT1 isoforms: A, p<0.05 (*), B, p = 0.3178 (ns), C, p = 0.0652(ns), and D, p<0.05(*) was performed, and RNA found to be more abundant in a Kaposi Sarcoma compared to tonsil tissue, using two-sided, unpaired student t-tests. Data shown is representative of two independent experiments performed in triplicate. **C.** RT-qPCR of viral genes, LANA, vFLIP, and K8.1 was performed and is shown. Statistical significance was determined by one way ANOVA, Tukey’s multiple comparisons test in iSLK-BAC16 with and without lytic induction compared to KS tissue, p<0.05(*), p<0.01(**), p <0.001(***), p <0.0001(****).

Lastly, to determine if WT1 expression is limited to cutaneous KS or also seen in other sites, we performed immunohistochemistry for LANA and WT1 in three cases of KS involving lymph nodes. There was clear positivity for WT1 in areas mirroring LANA expression (**Fig 2A**).

### Major oncogenic WT1 isoforms are upregulated in KS tumors

We sought to determine if the major WT1 isoforms overexpressed in leukemias and multiple solid tumors, which occur as a result of alternative splicing at Exon 5 and Exon 9 (WT1 isoforms A [EX5-/KTS-], B [EX5+/KTS-], C [EX5-/KTS+] and D [EX5+/KTS+] [24] are also overexpressed in primary KS lesions. To obtain high quality RNA we selected a representative case for which frozen tissue was available. Using RT-qPCR we demonstrated that RNA corresponding to the major oncogenic WT1 isoforms was more abundant in the KS-involved lymph node than in a control lymph node (**Fig 2B**), specifically isoforms A and D. We confirmed overexpression of total WT1 in an additional KS specimen using RT-qPCR, and in addition isoforms C and D. RT-qPCR for viral genes, including latent (LANA and vFLIP), as well as lytic (K8.1), was performed to assess viral gene expression. As controls, we used an *in vitro* model of viral latency, iSLK BAC-16, which can be induced to undergo lytic reactivation by treatment with 1mM sodium butyrate (NaB) and 1μg/ml doxycycline (doxy) (**Fig 2C**). Latent KSHV genes, vFLIP and LANA were detected in the KS tissue. K8.1 expression was increased in the iSLK BAC-16 treated with 1mM sodium butyrate and 1μg/ml doxycycline, but expressed at low levels in the tissue involved by KS, most consistent with a largely latent state in vivo expressing increased levels of WT1 and oncogenic isoforms compared to lymph node control tissue.

### WT1 is upregulated by KSHV infection

**Fig 3:**
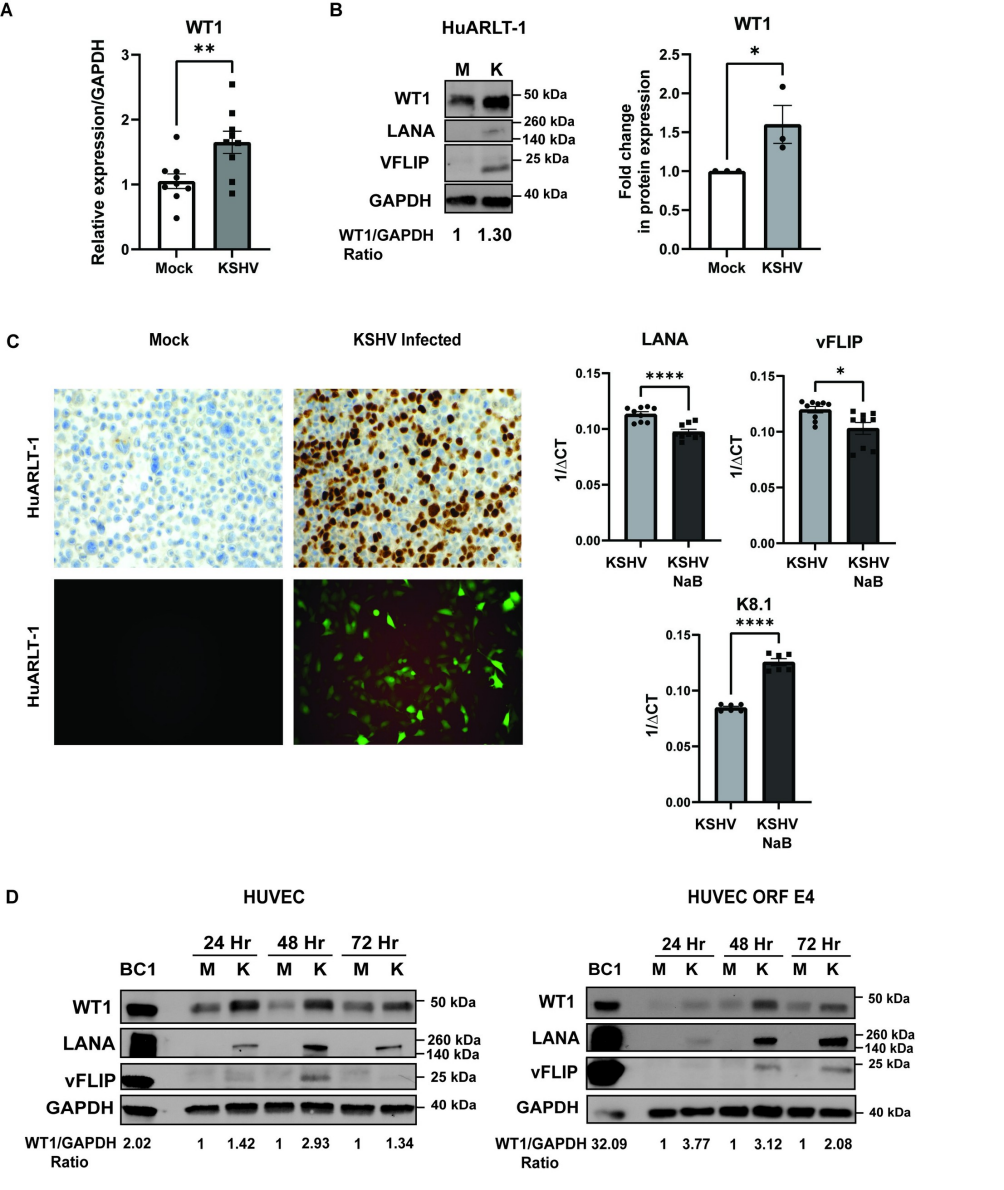
WT1 is upregulated upon de novo *in-vitro* KSHV infection. **A.** RT-qPCR demonstrating mRNA levels of WT1, p<0.01(**) in mock and KSHV-infected HuARLT-1 cells. **B.** Western blot of WT1, LANA, vFLIP upon KSHV infection of HuARLT-1 and corresponding densitometry analysis from 3 independent experiments shown of WT1/GAPDH ratio, p<0.05(*), using a one-sided unpaired t-test. **C**. (Top panel) Cell blocks of Mock vs. KSHV infected HuARLT-1, demonstrating IHC for LANA (nuclear brown), documenting infection. (Lower panel) Representative images of monolayer of mock vs KSHV infected HuARLT-1, obtained under fluoroscopy for GFP, indicative of KSHV BAC-16 infection in HuARLT-1 cells. Right panels show RT-qPCR mRNA levels of untreated and after lytic induction with 1mM Sodium butyrate (NaB) for LANA, p<0.0001 (****), vFLIP, p<0.05(*) and K8.1, p<0.0001(****), using two-sided, unpaired student t-tests. **D.** Western blots of WT1, LANA and vFLIP in primary HUVEC and HUVEC ORFE4 from 24 hours to 72 hours showing higher levels of WT1 in the setting of KSHV infection. Image densitometry analysis is shown below as the WT1/GAPDH ratio.

Given our findings of WT1 upregulation in KS tissues, we tested whether de novo KSHV infection of primary and immortalized endothelial cell lines directly leads to WT1 upregulation. After *in vitro* KSHV infection of the HuARLT-1 endothelial cell line, a conditionally immortalized human endothelial cell line [57] carrying doxycycline-dependent cassettes for autoregulated expression of the SV40 Tag and hTert, we demonstrate that WT1 is upregulated at the mRNA and protein levels (**Fig 3A** and **3B**). Cell blocks were made documenting LANA expression by IHC (**Fig 3C top panel**) or GFP expressed by KSHV-BAC-16 (**Fig 3C bottom panel**). The de novo KSHV infections performed demonstrated latent viral gene expression, vFLIP and LANA (**Fig 3C right panels**) at 72 hours. We also assessed for lytic gene expression based on amplification for K8.1, which was low compared to sodium butyrate treated cells at 72 hours. We further assessed whether our *in vitro* cultures, under the conditions and timing used in our study, represented latent or lytic infection (or a combination). Flow cytometry was performed since the BAC-16 used is a dual reporter virus, where mCherry is expressed in lytic cells (from ORFK4), and GFP is expressed constitutively, reflecting overall infection. There was no evident mCherry expression in the KSHV infected cells, while there was a clear mCherry positive population in 6–8% of cells that were treated with sodium butyrate (**S1 Fig**). We conclude that our cultures, under the conditions and times used for these studies, are largely latent and reflective of KS tissues. WT1 upregulation was confirmed in primary endothelial cells and a second cell line, by performing time course infections followed by WT1 protein analyses in primary HUVEC and HUVEC ORFE4 cells, a HUVEC line immortalized with adenovirus E4 [58] (**Fig 3D** left and right panels respectively). *In vitro* infection usually occurred in less than 40% of the total cellular population, so levels of WT1 seen by Western blot likely underestimate upregulation in the infected cells. Corresponding densitometry analyses demonstrated close to 3-fold upregulation of WT1 expression in comparison to GAPDH within 24 to 48 hours of KSHV infection (**Fig 3D)**.

### The latent viral oncoprotein vFLIP upregulates oncogenic isoforms of WT1 *in-vitro*

**Fig 4:**
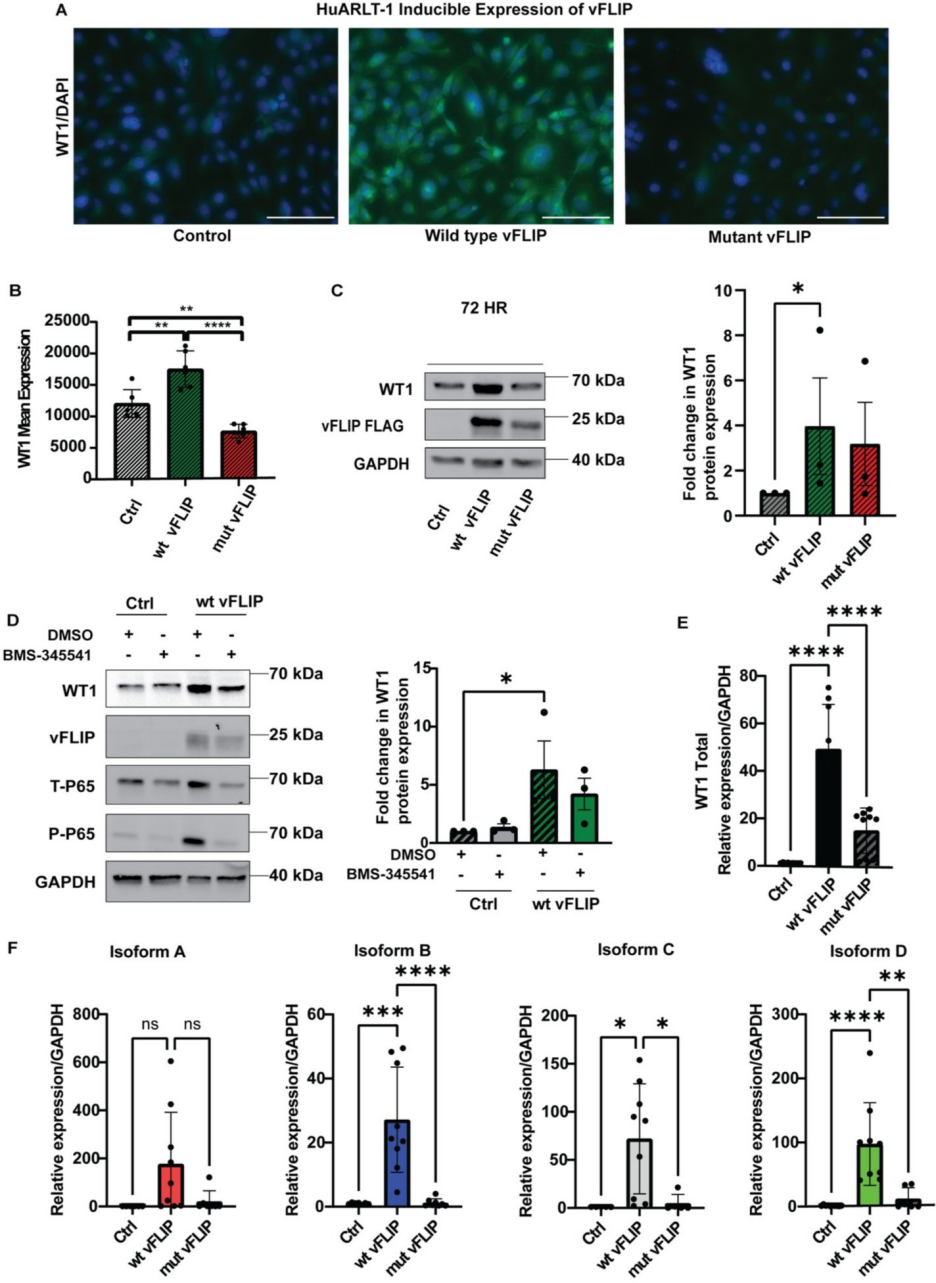
vFLIP upregulates major oncogenic WT1 isoforms. **A.** vFLIP expression upregulates WT1, demonstrated by immunofluorescence (green) in HuARLT-1 cells transduced with a doxycycline inducible pLVX vFLIP-FLAG lentivirus for wild type vFLIP vs mutant vFLIP compared to untransduced control cells. A mutant vFLIP that is unable to bind IKKγ and induce NFκB is defective in its ability to upregulate WT1. **B**. Quantitation of WT1 immunofluorescence (Arbitrary units) upon vFLIP induction, performed by averaging the GFP fluorescence for six distinct areas, showed increased WT1 expression with wild type vFLIP compared to control (HuARLT-1 not transduced with vFLIP), and mutant vFLIP: control vs. vFLIP. Statistical significance was determined using one way ANOVA, Tukey’s multiple comparisons, p<0.01(**), p<0.0001(****) **C.** Western blots show that WT1 protein is increased upon vFLIP-FLAG induction in HuARLT-1 cells. EMD Millipore WT1-NT antibody (clone 6F-H2) against WT1 was used. This increase was not seen to the same extent when the mutant vFLIP-FLAG is expressed, which is also less stable than the wild type form, as seen with antibodies to FLAG. Quantification of three independent experiments is shown, p = 0.05(*), using one-sided, student’s t test. **D.** Inhibition of NFκB signaling with BMS-345541 demonstrated reduction of WT1 expression, with quantification for WT1 in three independent experiments shown below. **E.** RT-qPCR for WT1 showed induction of WT1 mRNA with wild type vFLIP expression, which did not occur to the same extent upon mutant vFLIP expression: control vs. vFLIP, p<0.0001(****) and vFLIP vs. mutant vFLIP,p<0.0001(****) using two-way ANOVA with Tukey’s multiple comparison’s tests. **F.** Major WT1 isoforms, A, B, C, D are all found to be upregulated by RT-qPCR for WT1 *in vitro* upon vFLIP induction in HuARLT-1 cells, p<0.05(*), p<0.01(**), p <0.001(***), p<0.0001(****), not significant (ns), using ordinary one way ANOVA, Tukey’s multiple comparisons test.

By virtue of its ability to induce NFκB, a transcription factor known to regulate WT1 levels [50], the KSHV protein vFLIP [48,49] may regulate WT1 expression. To investigate this possibility, HuARLT-1 cell lines were transduced as previously described [59] with a doxycycline-inducible pLVX vFLIP-FLAG lentivirus. In parallel, cells were transduced with an NFκB-dead mutant (vFLIP^AAA(58–60)^) that renders vFLIP unable to bind IKKƴ. Stable lines were established with puromycin selection. WT1 was upregulated upon wild type vFLIP induction, but not with mutant vFLIP (**Fig 4A, 4B and 4C)**. The observation that vFLIP protein levels were lower with mutant vFLIP, is consistent with an NFκB-dependent mechanism, but this result should be interpreted with caution, because this mutation also destabilizes vFLIP, leading to lower expression. It nevertheless confirms that vFLIP on its own can induce increased expression of WT1. To investigate whether inhibiting NFκB signaling would impact WT1 expression, we treated HuARLT-1 cells with induced vFLIP expression with BMS-345541, an NFκB inhibitor, at 24 hours compared to DMSO controls, and demonstrated similarly a loss of WT1 expression (**Fig 4D**). Upon vFLIP induction, total and phospho-p65 levels were initially increased and then upon treatment with BMS-345541, these levels diminished. WT1 upregulation upon vFLIP induction was also demonstrated at the RNA level by RT-qPCR in HuARLT-1 cells, where WT1 levels were increased with wild type vFLIP, more so than with mutant vFLIP (p<0.0001; (**Fig 4E**). We then performed RT-qPCR [24] to identify the major WT1 isoforms which occur as a result of alternative splicing at Exon 5 and Exon 9. vFLIP induction in HuARLT-1 led to upregulation of major WT1 isoforms A, B, C, D and total WT1 within the wild type vFLIP line compared to the mutant vFLIP (**Fig 4F**). Confirmation of vFLIP expression by RT-qPCR is shown in **S2 Fig.**

### siRNA targeting of WT1 in KSHV infection decreases cell proliferation, pAKT, and BCL2 expression

**Fig 5:**
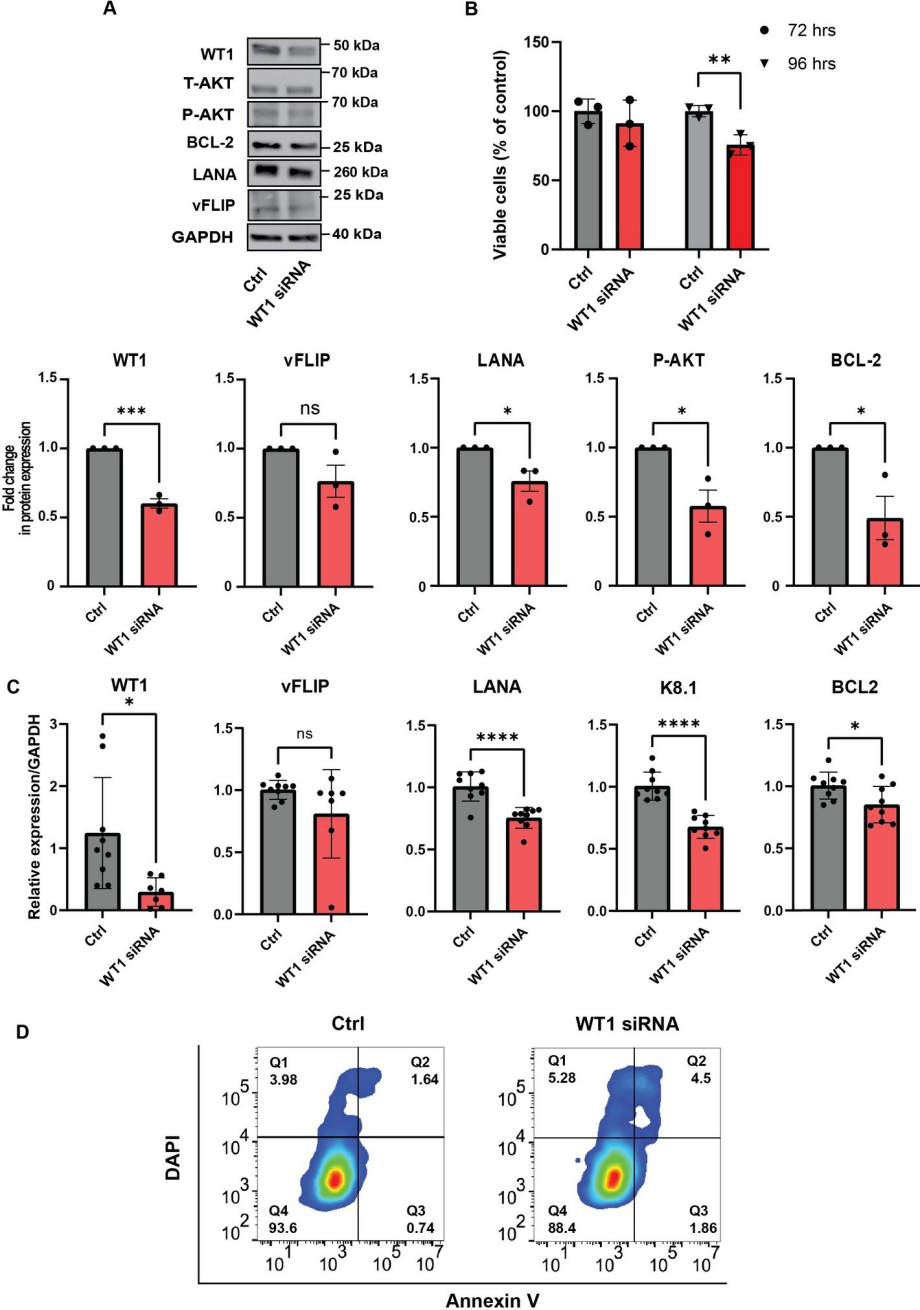
WT1 siRNA decreases proliferation, pAKT, and Bcl2 expression. **A.** Western blotting demonstrates WT1 knockdown in iSLK KSHV BAC-16 cells transfected with WT1 siRNA in comparison to a control siRNA. Corresponding densitometry analysis is noted as protein fold expression compared to GAPDH for WT1, p<0.001(***), vFLIP(ns), LANA p<0.05(*), pAKT p<0.05(*), and BCL2 p <0.05(*) using two-sided, unpaired student t-tests. **B.** Introduction of WT1 siRNA leads to decrease total cell number using trypan blue to assess cell viability compared to control siRNA, p<0.01 (**) using two-sided, unpaired student’s t-tests. **C.** RT-qPCR of WT1, p<0.05(*), vFLIP(ns), LANA, p<0.0001(****), K8.1, p<0.0001(****), and BCL2 p<0.05(*) in the setting of WT1 knockdown in ISLK BAC-16 with WT1 siRNA in comparison to a control siRNA using two-sided, unpaired student’s t-tests. **D.** iSLK-KSHV BAC-16 cells transfected with WT1 siRNA compared to control siRNA, demonstrating flow cytometry for DAPI and Annexin V revealing increased apoptosis, performed in duplicate.

Previous studies in various cancer cell lines demonstrated that loss of WT1 results in decreased proliferation by interfering with antiapoptotic functions. The critical role of WT1 in programmed cell death has been at times conflicting, depending on the cellular context, with differing impacts on the antiapoptotic gene family, BCL2 [60], suggesting that the role of WT1 as a tumor suppressor or oncogene could be a result of WT1 effects on BCL2 family members. In a lung cancer model, a positive feedback loop was demonstrated between the WT1 and the PI3K/AKT signaling pathway, and loss of WT1 led to decreased proliferation and loss of pAKT as well as decreased expression of antiapoptotic BCL2 [61]. AKT and BCL2 are known to be upregulated upon de novo KSHV infection [62–64]. We transfected iSLK cells that contain the KSHV-BAC16 genome with WT1 siRNA, in parallel with a control siRNA. Transfection of WT1 siRNA resulted in decreased WT1 protein expression (**Fig 5A**) associated with decreased cell viability (**Fig 5B**) as compared to the control siRNA. Decreased WT1 protein expression was accompanied by decreased protein levels of phosphoAKT, but not total AKT (**Fig 5A**) suggesting a role for WT1 in AKT signaling. In addition, there was a decrease in BCL2 and LANA protein levels. Partial loss of WT1 was also noted at the mRNA levels, associated with a corresponding decrease in LANA and K8.1 mRNA levels (p<0.0001 and p<0.0001, respectively) (**Fig 5C**). Of note, there was a decrease in BCL2 mRNA levels (p<0.05, as seen by RT-qPCR (**Fig 5C**). Consistent with the decrease in BCL2 expression, there was noted evidence of increased apoptosis with WT1 siRNA in iSLK-BAC16 cells compared with the control iSLK-BAC16 cells (**Fig 5D**).

### WT1 is inversely correlated with T cells in the tumor microenvironment

**Fig 6:**
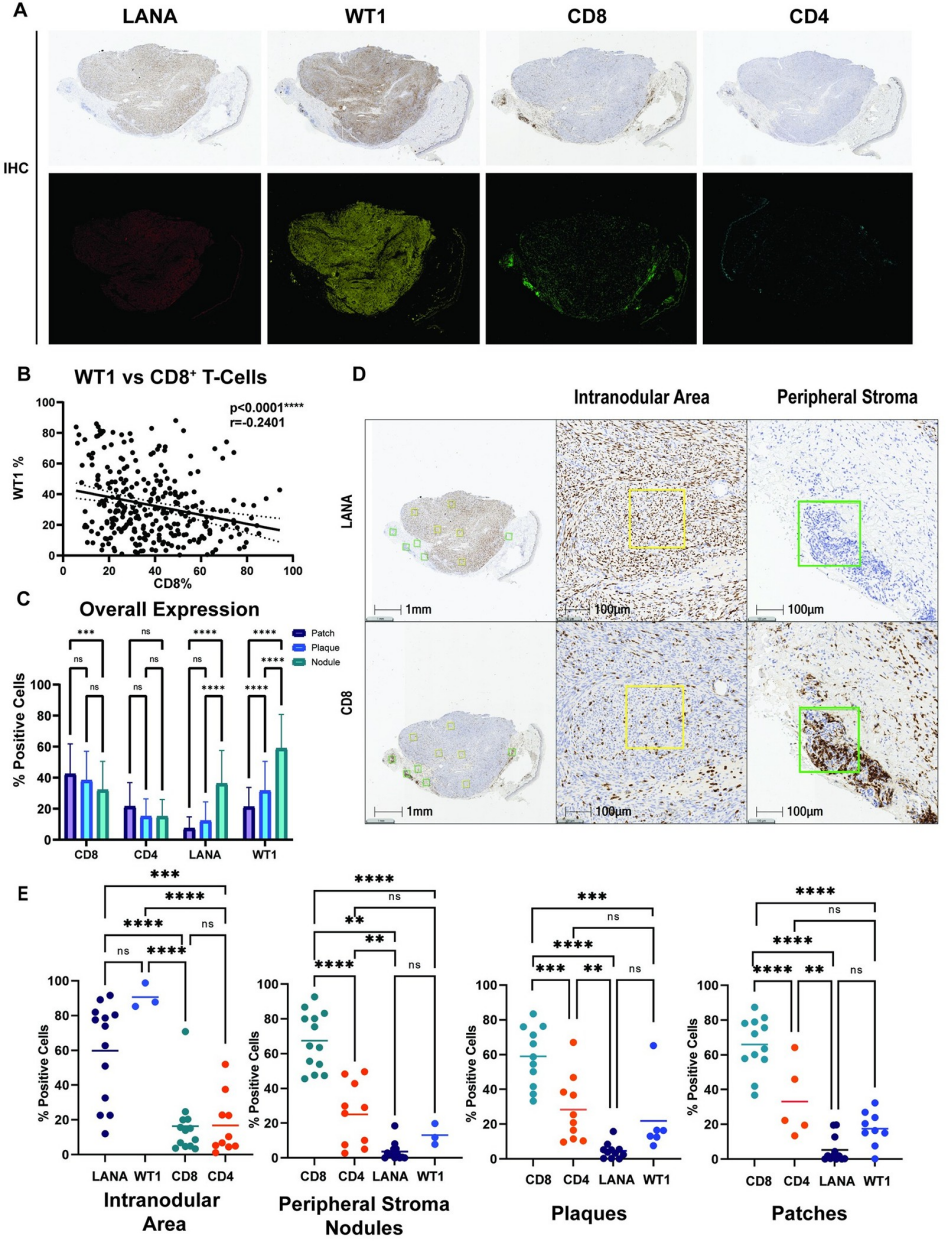
WT1 and LANA expression correlate with decreased T cell infiltrates. **A.** Using HALO analysis software of KS tumors from the AMC066/A5263 clinical trial, pseudocolor images were created for IHC of LANA, WT1, CD8, and CD4 cells. **B.** WT1 expression in all cases was inversely correlated with the number of CD8+T cells, using Spearman’s correlation test. Of note, melanin is noted along the epidermis and was excluded upon analysis of WT1 expression. **C.** Overall assessment of cellular populations according to histological subtype showed lower CD8 and CD4 T cells with advanced histological subtype, and the reverse for WT1 and LANA, p <0.001(***), p <0.0001(****) using two way ANOVA, Tukey’s multiple comparisons test. **D.** Clusters of CD8+ cells were seen at the periphery of nodular KS lesions, a representative case. **E**. Areas with numerous LANA+ cells were selected and quantified for CD8 positivity in a sequential tissue section, and vice versa using ordinary one-way ANOVA, Tukey’s multiple comparisons test. This spatial analysis was performed for WT1, LANA, CD4+T cells and CD8+T cells in areas of high LANA (intranodular areas) or high CD8 +T cells (along the periphery of nodular lesions), and lymphocytic infiltrates in corresponding regions for WT1/LANA/CD8/CD4 in plaques and patches. Ordinary one-way ANOVA multiple comparisons was performed as shown in **S4 Table**, p<0.05 (*), p <0.01 (**), p <0.001(***), p <0.0001(****), ns (not significant).

Given that previous studies suggest that WT1 may play a role in regulating the presence of immune cells in the tumor microenvironment [30], the spatial relationship between expression of WT1 and LANA and immune cell infiltrates was examined. In general, few B cells were seen, and plasma cells ranged from very abundant, sometimes in large clusters, to sparse. In general, CD8+ T cells were more abundant than CD4+ T cells. These were present in areas rich in inflammatory cells, which were usually concentrated at the margins of regions involved by KS (LANA+ and WT1+). This was more evident in nodular lesions, where KS areas are well defined (**Fig 6A** top panel). CD8+ and CD4+ T cells were also present within areas containing sheets of spindle cells in KS nodules, but these were sparse and dispersed, rather than forming inflammatory aggregates as seen at the periphery of these lesions.

To formally assess a possible correlation between WT1 and CD8 expression in KS lesions we conducted image analysis using HALO software to create pseudocolor images of IHC for LANA, WT1, CD8 and CD4 (**Fig 6A** lower panel). Overall correlation analysis revealed an inverse relationship between WT1 expression and CD8+T cells (r = -0.25; p<0.0001; **Fig 6B**). In examining the distribution of expression of WT1, LANA, and CD4, and CD8 T cells, lower CD8 and CD4 T cells were seen with advanced histological subtype, and the reverse for WT1 and LANA (**Fig 6C**). Thirteen nodules, eleven plaques and twelve patches were evaluable as sequential sections and analyzed by selecting five corresponding high LANA and high CD8+ T cell abundant areas for each lesion (**Figs 6D–6E and S3A–S3C**) performing comparisons as previously described [65]. Focusing on areas with high WT1+ and LANA+ cells within KS nodules, there were notably lower percentages of CD8+T cells and CD4+T cells in these regions (LANA = 60% vs. CD8 = 16%, p<0.001; LANA = 60% vs. CD4 = 17%, p = 0.0002; WT1 = 91% vs. CD8 = 16%, p<0.0001; and WT1 = 91% vs. CD4 = 17%, p<0.0001), whereas in areas of low WT1+/LANA+ cells immediately adjacent to nodules in the peripheral stroma, there was a higher percentage of CD8+T cells (CD8 = 67% vs LANA = 4%; p<0.01 and CD8 = 67% vs WT1 = 13.11%, p<0.0001) (**Figs 6E and S3A**). Analyses on plaques and patches are shown in **Fig 6E** (two right panels). Statistical analysis for these lesions is shown using one way ANOVA, Tukey’s multiple comparisons tests in **S4 Table.** Representative cases for the plaque and patches are shown in **S3B–S3C Fig**. Taken together, these findings indicate that lymphocytic aggregates that are rich in T cells localize outside the KS nodules in nodular lesions, and are consistent with a relatively immunosuppressive immune environment in the immediate proximity of the KSHV-infected spindle cells that have higher percentages of WT1+ and LANA+ cells.

### WT1 is a potential target for immunotherapy in Kaposi sarcoma

**Fig 7:**
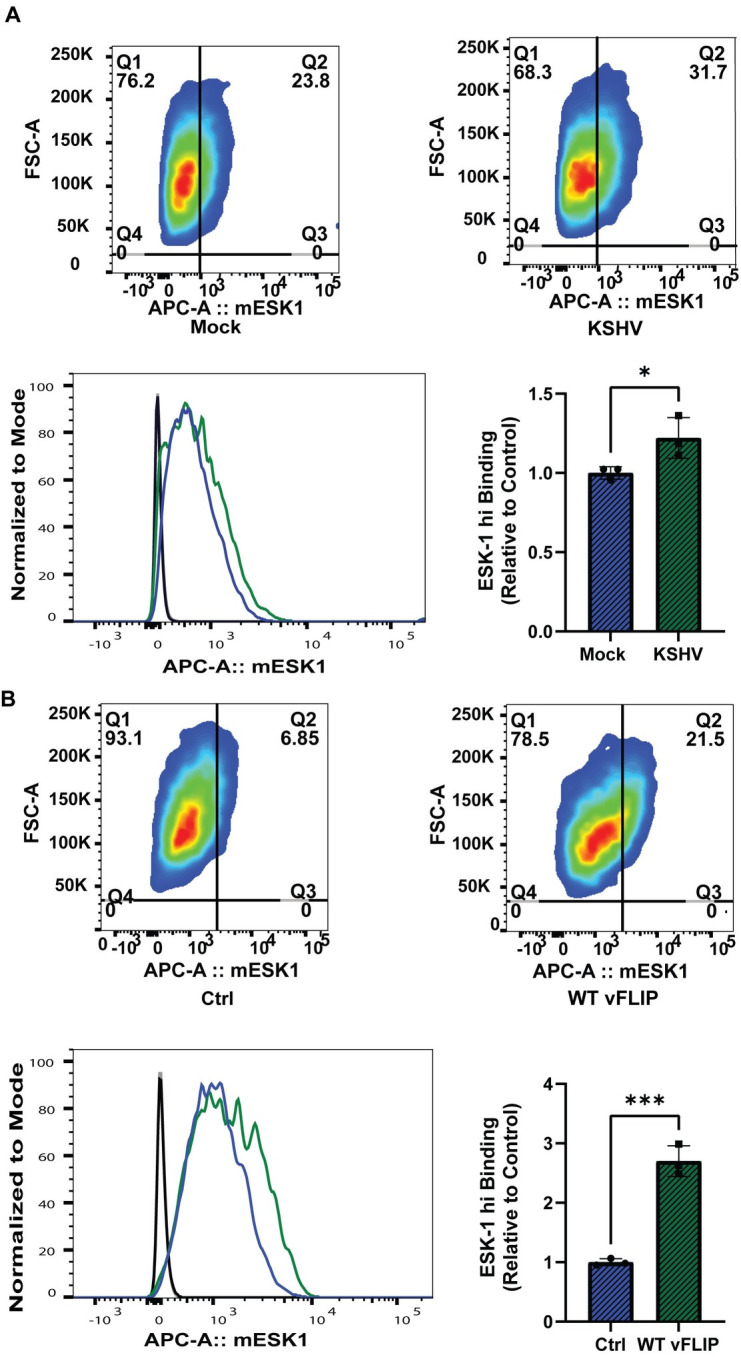
WT1 is a potential target for immunotherapy in Kaposi sarcoma. **A**. ESK-1 binding assays with HLA-A0201 transduced HuARLT-1 cells at 6 days post KSHV infection (green) or mock infection (blue) and ESK-1 antibody incubation compared to IgG isotype controls (black line), assessed through flow cytometry (*p< 0.05) using two-sided, unpaired student t-tests. **B.** ESK-1 binding assays performed with HLA-A0201 transduced HuARLT-1 cells, comparing control (blue) vs. ‘vFLIP’ (green) at 96 hours of doxycycline induction of vFLIP expression and ESK-1 antibody incubation compared to IgG isotype controls (black line) left panel, assessed through flow cytometry (***<0.001) using two-sided, unpaired student’s t-tests.

The effect of KSHV infection on binding of ESK-1 antibodies, which recognize WT1 peptides presented on HLA-A0201 [36], was tested by transducing HuARLT-1 with HLA-A0201, followed by KSHV infection which resulted in infection of approximately 30% of cells measured by GFP expression. Subsequent incubation with ESK-1 performed in triplicate showed 23% positivity in mock-infected cells vs 28% in KSHV infected cells (p<0.05; **Fig 7A**), a 1.2 fold increase in binding assessed by flow cytometry. Upon doxycycline treatment of HuARLT-1 with HLA-A0201 transduced with a doxycycline inducible pLVX vFLIP-FLAG lentivirus, ESK-1 binding performed in triplicate increased from 7.2% to 19.4% (p<0.001) (**Fig 7B**), a 2.7 fold increase in binding, consistent with the hypothesis that vFLIP expression alone upregulates WT1 and increases ESK-1 binding. There was no change in overall viability, consistent with this antibody not being directly cytotoxic in the absence of WT1-directed T cells [36]. The values represent the average of 3 separate experiments, gating upon FSC-A and ESK-1 APC positive cells for each set of experiments.

### Baseline WT1 expression did not correlate with clinical outcomes in individuals with advanced stage AIDS/KS in response to combined antiretroviral therapy with chemotherapy

The largest cohort of KS biopsies evaluated for WT1 expression was from a three arm open-label, randomized, non-inferiority trial of PLWH and pathologically confirmed advanced stage KS collected as part of a joint AMC and ACTG clinical trial AMC066/A5263 (NCT01435018) [54]. This study compared the safety and efficacy of three chemotherapy regimens (oral etoposide (ET), bleomycin and vincristine (BV), or paclitaxel (PTX) together with ART (Efavirenz/Emtricitabine/Tenofovir Disoproxil Fumarate). PTX+ ART showed superiority to both ET +ART and BV+ ART with respect to the primary outcome, week-48 progression-free survival. Trial participants were categorized post hoc in to the following groups based on their overall response to KS as follows: Good responder (response that is complete or partial by week 12 that lasted for a minimum of 24 weeks), progressor (evidence of progression without prior response, or progression after an initial response that was less than 24 weeks duration), and stable (stable response for a minimum of 24 weeks with neither response nor progression on study). The distribution of WT1 positive cells was similar for good responders and progressors (p = 0.52, **S5 Table**). We did not find any evidence that WT1 expression in the biopsies correlates with clinical outcome in this group with advanced stage KS in response to combined antiretroviral therapy with cytotoxic chemotherapy.

## Discussion

This study shows that WT1 is overexpressed in the vast majority of Kaposi sarcoma lesions and is largely absent in adjacent normal tissue. Importantly, WT1 expression is highest in cases with histopathologically advanced KS, i.e., nodules. We found that total WT1, in addition to isoform A and D were upregulated in KS tissue. This finding is consistent with prior studies demonstrating upregulation of oncogenic WT1 isoforms known to be overexpressed in solid tumors and leukemias [24,66–68] and associated with multiple oncogenic functions including promotion of proliferation, antiapoptotic activity, angiogenesis and metastases [32,33,66]. Our findings also show somewhat higher WT1 expression in HIV-associated KS than HIV-negative KS lesions, even when lesions with similar histologies are compared. This observation raises the possibility that HIV may impact WT1 expression directly or indirectly. An important impact of HIV infection, especially if uncontrolled, is the dysregulation of both the adaptive and innate host immunity, leading to both immunodeficiency and chronic inflammation with notable increase in inflammatory cytokines [69], which may contribute to upregulation of WT1, perhaps through activation of transcription factors, such as NFκB, known to regulate WT1 transcription. Other potential factors contributing to higher WT1 expression in PLWH could be HIV-1 proteins such as HIV1 encoded Tat, a small cationic peptide that can enter multiple different cells [70], and promote KSHV infection, angiogenesis, and cooperate with KSHV proteins to impact multiple signaling pathways [24,32,33,66–68,71].

Clearly evident from our studies is that KSHV can upregulate WT1 in endothelial cells in latently infected cells. KSHV infection *in vitro* increases WT1 expression at both the mRNA and protein levels. In KS lesions, KSHV is largely latent, expressing only a handful of viral gene products. Our *in vitro* infection cell culture assays are also largely latent, reflective of KS lesions. Examination of a potential role for WT1 during the viral life cycle, and in particular during lytic replication, is ongoing. Here we demonstrate that one of the KSHV latent genes, vFLIP, also increases WT1 expression, upregulating major oncogenic WT1 isoforms, and indicating that this viral protein is at least partly responsible for KSHV-mediated WT1 upregulation. Several signaling pathways are known to control WT1 expression, one of which is NFκB [50]. We show that a NFκB dead mutant vFLIP was not capable of upregulating WT1 levels, suggesting vFLIP upregulates WT1 at least partly through NFκB activation, which was confirmed using an NFκB inhibitor.

We achieved partial decrease of WT1 protein and mRNA levels using RNA interference, although methods that would lead to total WT1 elimination, like CRSPR/Cas9 were unsuccessful, which suggests that at least low WT1 expression in KSHV-infected endothelial cells in culture is essential for cellular survival. Nevertheless, this partial decrease led to decreased total numbers of cells, increased total cell death with evidence of increased apoptosis. In addition, WT1 knockdown led to decreased phosphorylation of AKT, consistent with reported effects in other cancer models [61]. We also saw decreased levels of BCL2 protein at the protein and mRNA level. These findings support a potential oncogenic role of WT1 in KS, as demonstrated in many previous studies of solid tumors and leukemias [66,72] consistent with the pro-survival/proliferation functions of WT1.

Analysis of KS tumor specimens using immunohistochemistry also revealed that there is an important microenvironmental impact associated with WT1 expression. While upregulation of WT1 in KSHV-infected endothelial cells *in vitro* was consistent among multiple experiments and different endothelial cell types, WT1 levels appear to be much higher *in vivo*. Moreover, WT1 expression in tissue biopsies significantly correlated with the expression of the viral latent oncoprotein LANA, and inversely correlated with T cells within the tumor and surrounding stromal tissue. Further studies are needed to investigate the heterogenous expression of WT1 and LANA, at times colocalizing in KS cases and at other times in separate but neighboring cells.

The low T cell infiltration observed in areas of high WT1 and LANA suggests that WT1 may contribute to the immunosuppressive functions of KSHV in creating an immunosuppressive tumor microenvironment. In fact, one study found that knockout of WT1 in mouse endothelial, hematopoietic and myeloid derived suppressor cells (MDSCs), led to decreased tumor growth and metastases, and that WT1 was critical to recruiting MDSCs to suppress T cell immune responses [30]. In addition, our observations are consistent with a recent study that demonstrated decreased immune infiltrates in areas of KS lesions showing abundant KSHV-infected cells compared to surrounding areas [65]. While both CD4+ and CD8+ T cells are also present among the spindle cells, their functional phenotypes remain to be characterized. Thus, ongoing studies are aimed at further defining the immune components of KS lesions, including further characterization of functional T cells subsets (i.e. activation, exhaustion, Tregs, memory), as well as other cells, such as tumor associated macrophages (TAMs) and MDSCs. We have previously generated transgenic mice expressing vFLIP in endothelial cells. This was associated with remodeling of myeloid differentiation with M1 toward M2 polarization, and expansion of MDSCs and TAMs [48]. These *in vivo* observations are consistent with a model whereby vFLIP induces WT1, which in turn recruits MDSCs to the tumor microenvironment. Furthermore, WT1 has been shown to induce a number of other factors [72–75] that can promote tumorigenesis and angiogenesis and have been shown to be expressed by KS spindle cells or KSHV infected endothelial cells in culture, including BCL2, MMP9, VEGF and PDGF receptors [76–80].

We also investigated whether a TCR mimic antibody against WT1 and HLA-A0201 would bind endothelial cells with increased WT1 expression as a result of KSHV infection or vFLIP induction. We found that both conditions increased ESK-1 binding compared to their control counterparts. These findings suggest that WT1 directed immunotherapies, ESK1 [36], or with bi-specific T cell-engaging antibodies (BiTEs) [81] or anti-WT1 T cells [82] or CAR T cells [83,84] may have therapeutic potential in KS. Other immunotherapeutic approaches include WT1-directed peptide vaccines, which have been studied in phase 1 and phase 2 trials for hematologic and solid malignancies [37–39]. Given that WT1 peptide vaccines have been found to be safe and induce clinical and immunological responses [37,85], this approach may be attractive for use as a therapeutic vaccine against KS, especially in chemotherapy-resistant or recurrent cases as adjunctive therapy or in low grade disease which may be controlled without the need for chemotherapy.

Lastly, WT1 expression may serve as a biomarker to identify cases most likely to respond to WT1 immunotherapy, which may be especially important in cases of advanced KS that disproportionately affect PLWH. While our study did not demonstrate an association of baseline WT1 expression with the response of advanced KS to cytotoxic chemotherapy, it is important to note that these individuals all had HIV AIDS and had received little to no prior ART treatment prior to enrollment in the study [54]. In addition, lesions from one individual patient can be highly heterogeneous, and we only examined the diagnostic biopsy from each patient, that may not be reflective of the overall disease burden. Nonetheless, WT1 expression was found to correlate with more advanced histopathologic subtype. A follow up study comparing early to late stage KS, while evaluating WT1 expression at baseline and in follow up after ART alone or in combination with directed KS treatment, may be able to better assess whether WT1 may have the potential to serve as a biomarker of treatment responses.

**Fig 8:**
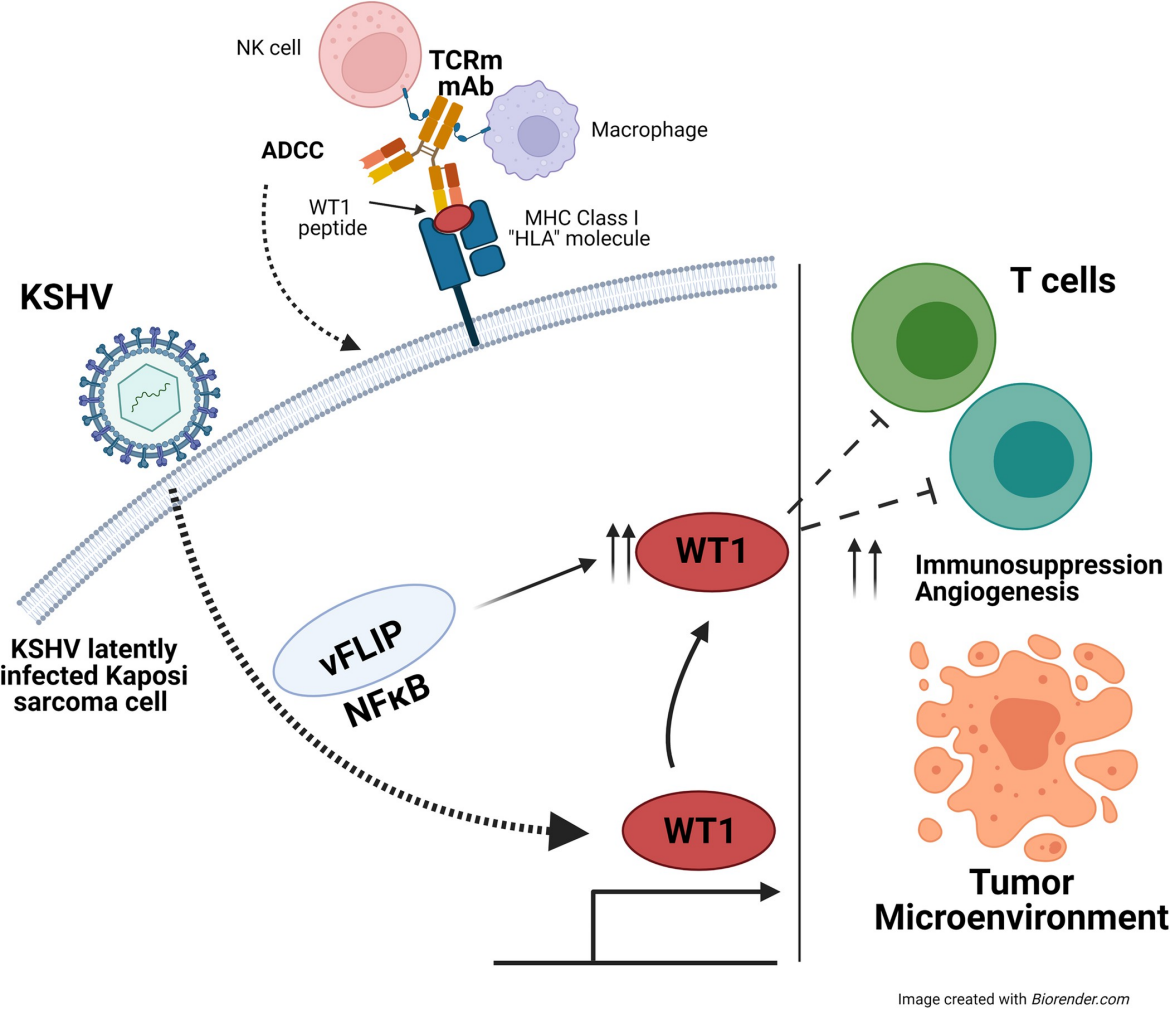
Schematic of a Proposed Model of WT1 Mediated Tumorigenesis in KS and WT1 ESK-1 Immunotherapy. A diagram demonstrating KSHV infection contributing to vFLIP mediated upregulation of WT1 through NFκB activation. WT1 upregulation in combination with KSHV, potentially impact the tumor microenvironment, contributing to an immunosuppressed state, in the suppression of T cells. WT1 directed immunotherapy such as TCRm mAb may aid in reversing the effects of WT1 mediated tumorigenesis through the targeting of KS spindle cells overexpressing WT1 through the induction of antibody-dependent cellular cytotoxicity (ADCC). (*Image created with Biorender*.*com)*.

Taking our findings together, we propose a model of WT1 mediated tumorigenesis in Kaposi sarcoma (**Fig 8**) in which following KSHV infection, latent viral genes are expressed, including vFLIP, which activate NFκB, leading to upregulation of oncogenic WT1 isoforms. In turn, WT1 expression, in conjunction with expression of other viral genes, leads to an immunosuppressive and angiogenic tumor microenvironment. An implication of our observations is that immunotherapy directed towards WT1 in KS may target KS spindle cells overexpressing WT1 and potentially aid in reversing the immunosuppressive tumor microenvironment. This study highlights the overexpression of WT1 in advanced KS cases and a mechanism of KSHV vFLIP- mediated WT1 upregulation and underscores the potential for immunotherapy directed against WT1 as a novel strategy to treat Kaposi sarcoma.

## Materials and methods

### Ethics statement

The Weill Cornell Medicine (WCM) Institutional Review Board approved the use of specimens both archived at WCM and those sent to the Cesarman Laboratory at WCM from other institutions. Tissue specimens from Makerere University of Kampala Uganda were also approved for use from the School of Biomedical Sciences Higher Degree Research and Ethics Committee of Makerere University. Tissue specimens from the Stroger Hospital of Cook County were obtained also under the IRB of Cook County Health & Hospitals System. Specimens obtained from the AMC/ACTG study, were provided with participants’ written informed consent from each trial site in a multinational clinical trial (ACTG A5263/AMC 066; NCT01435018) [54]. For the additional specimens used, WCM IRB does not require consent for the use of residual archived tissue specimens that have been anonymized.

### Participant specimens

KS biopsies were obtained from multiple cohorts. The largest included 334 diagnostic KS tumor biopsies obtained as part of an open-label, randomized, non-inferiority clinical trial in adult PLWH with advanced, previously untreated, biopsy-confirmed KS, conducted by the AIDS Malignancy Consortium (AMC) and AIDS Clinical Trials Group (ACTG), AMC066/A5263 (NCT01435018), and conducted at 11 sites in sub-Saharan Africa and Brazil [54]. A total of 294 specimens of KS biopsies were evaluated. Participants were enrolled between 2013 and 2018 and were randomized to receive one of three chemotherapy regimens together with standard antiretroviral therapy [54]. A second cohort comprised 23 KS biopsies from PLWH from the Infectious Disease Institute of Makerere University in Uganda. A third cohort comprised 8 KS biopsies from patients with HIV-associated KS from Stroger Cook County Hospital in Chicago. A fourth cohort included 26 KS biopsies from the Department of Pathology and Laboratory Medicine of Weill Cornell/New York Presbyterian Hospital, half of which were from PLWH, and the remainder from individuals with no evidence of HIV infection or iatrogenic immunosuppression. For acquisition of frozen tissue sections, a Weill Cornell archive of KS tissue samples was sampled.

### Cells and reagents

iSLK cells that harbor wild-type KSHV BAC-16 [86,87] were used to make KSHV virus stocks and were cultured in Dulbecco’s modified Eagle’s medium (DMEM) (GIBCO, 11965–092) with 10% FBS (GIBCO, 10437028), 50 μg/mL gentamicin sulfate (Caisson Labs, ABL05), and selection antibiotics 1 μg/ml puromycin (Thermo Fisher Scientific, A1113803), 250 μg/ml G418 (Thermo Fisher Scientific, 10131035) and 1.2 mg/ml hygromycin B (Corning, 30-240-CR). In addition, the iSLK line were cultured in DMEM with 1 μg/ml puromycin and 250 μg/ml G418. The iSLK BAC16 were cultured in DMEM with selection antibiotics 1 μg/ml puromycin, 250 μg/ml G418, and 1,2000 μg/ml hygromycin B. The WT1 siRNA and control siRNA were obtained from Thermo Fisher. HuARLT-1 cells were cultured in endothelial cell growth medium EGM-2 (Lonza, CC-3162) with 2μg/ml of doxycycline (Sigma-Aldrich, D9891-1G). Human umbilical vein endothelial cells (HUVEC) (Lonza, C2517A) were cultured in EGM-2. HUVEC ORFE4, a kind gift from the lab of Shahin Rafii at Weill Cornell Medicine were cultured in EGM-2 and did not require selection. All primary cell cultures and cell lines have undergone testing for mycoplasma and were confirmed negative.

### Plasmids

HuARLT-1 cells were transduced with a gamma retroviral vector with HLA-A0201 packaged from 293T HEK. Stable HuARLT-1 cell lines expressing HLA-A0201 were sorted using an ARIA-2 flow cytometer within the Weill Cornell Medicine Cytometry Core. WT vFLIP and vFLIP NFκB dead mutant inducible cell lines were established using previously described doxycycline inducible lentiviral vectors [59]. Stable transduced cell lines were established in HuARLT-1 by puromycin selection at 1μg/ml and doxycycline 2μg/ml for inducible expression of WT vFLIP and vFLIP null NFκB.

### KSHV BAC-16 infection

KSHV BAC-16 stocks of virus were prepared from stable iSLK cells as previously described [87,88]. These studies utilized a BAC-16 clone, K4mCherry designed by Dr. Jennifer Totonchy in the lab of Dr. Ashlee Moses, which is a dual reporter, expressing GFP constitutively, indicative of KSHV infection, as well as containing a C terminal fusion mCherry to the K4 gene. Hence, in the manuscript, the referral to iSLK-BAC16 refers to the K4mCherry clone herein described, unless otherwise noted. Upon treatment with lytic inducing agents, doxycycline and sodium butyrate, mCherry is expressed, in addition to the constitutively active GFP. iSLK-BAC-16 were cultured in DMEM supplemented with 10% FBS, 50 μg/mL gentamicin sulfate, 1 μg/ml puromycin, 250μg/ml G418 and 1,2000 μg/ml hygromycin B. Stable iSLK-BAC16 cells were lytically induced with the addition of both doxycycline (1 μg/ml) and sodium butyrate (3 mM). Four days later, supernatant was collected and cleared of cells and debris by centrifugation (2,000 rpm for 5 min at 4°C) and filtration (0.45 μm). Virus particles were pelleted by ultracentrifugation (25,000 rpm for 1.5 h at 4°C) using an SW32 Ti rotor. A spinoculation with KSHV was performed on the respective cell line, using KSHV virus in the presence of 8μg/ml polybrene (Sigma-Aldrich, TR-1003) in serum-free media, at 1000rpm for 30 min at 4°C. Images were obtained at 20x using the Olympus BX63 Fluorescence Microscope with DP80 Camera.

### Lentiviral transduction

Lentiviral packaging was performed using Effectene (QIAGEN, 301427) per packing instructions with the packaging plasmids psPAX2 and vsvg into HEK293T cells. Supernatant containing lentivirus particles were collected at 48 and 72 hours and filtered (0.22 μm) and stored at -80°C. HuARLT-1 cells, and 293T cells underwent spinoculation, with filtered supernatant in the presence of 8μg/ml polybrene (Millipore-Sigma, TR-1003-G) in serum free media, at 1000rpm for 30 min at 4°C. Transduced cells were then selected using puromycin 1μg/ml.

### Western blotting

Total protein extracts were prepared from *in-vitro* 2D cell culture systems using RIPA buffer (Thermo-Fisher, 89900). Quantification of protein lysates was done using Pierce BCA assay (Pierce, 23225). Proteins were separated using 10% sodium dodecyl sulfate-polyacrylamide gel electrophoresis SDS-PAGE gel (BioRad, 1610772) and then transferred to a polyvinylidene difluoride membrane (PVDF) (GE Healthcare, 10600023) and blocked in 5% nonfat dry milk-TBST for 1 hour at room temperature. The PVDF membrane was then incubated overnight with primary antibodies diluted in 5% BSA-TBST overnight at 4°C and then immunoassayed using standard methods. The following primary antibodies were used for western blotting for WT1: rabbit anti-WT1 (Proteintech, WT1 12609-1-AP, 1:600) and mouse anti WT1-NT (EMD Millipore, clone 6F-H2, 05–753, 1:200). Western blotting for this study was mostly performed with the WT1 antibody from Proteintech, which preferentially demonstrated a prominent band at approximately 49kDa., unless as specified in the manuscript, the WT1 antibody from Millipore, was used, which in addition to the 49kDa. band demonstrated a more prominent band between 55 and 60kDa. as seen in **Fig 4C** and **4D**. The additional following primary antibodies were used: rat anti-LANA (Millipore LNA Anti HHV8, 1:1000), rabbit anti-Flag (Rockland, 600-401-383, 1:1000), rabbit anti-phospho AKT (Cell Signaling Technology, 1:1000), rabbit anti-total AKT (Cell Signaling Technology, C67E7, 1:1000), rabbit anti-phosopho p65 (Cell Signaling, clone 93H1, 1:500), rabbit anti-total p65 (Cell Signaling Technology, C22B4, 1:500), mouse anti Bcl-2 (Cell Signaling (124), 1:1000), rabbit anti-GAPDH (Genetex, 1:50,000), a rat anti-vFLIP (clone 4C1, 1:200). The following secondary antibodies were used for western blotting, rabbit anti-HRP (GE healthcare, NA9340V, 1:5000–1:7500), mouse anti-HRP (GE healthcare, NA931V, 1:2000–1:5000) and goat anti-rat IgG (H&L) HRP (Thermo Fisher Scientific, 31470, 1:5000).

### RT-qPCR

Total RNA was isolated according to Qiagen RNAeasy Mini kit (QIAGEN, 74106) standard protocols. RNA purity was confirmed with the ratio of the absorbance at 260 and 280nm using a spectrophotometer (Nanodrop). The reverse transcription reaction was adapted from protocols supplied by Applied Biosystems, using the High Capacity cDNA Reverse Transcription Kit, Thermo Fisher Scientific. Briefly, reactions were incubated for 10 minutes at 95°C followed by 50 cycles of 15 seconds at 95°C and 60 seconds at 60°C. All reactions were completed in triplicate. The quantitative RT-qPCR and fluorescence measurements were made using the Applied Biosystems 7500 Real-Time PCR System. B-actin and GAPDH were utilized as housekeeping genes for normalization to calculate the relative expression fold change for the target genes using the 2^(-ΔΔCT), unless otherwise stated. Primers used for qPCR are listed in **S6 Table**. Detection of the WT1 isoforms by qPCR have been previously validated for optimized primer design and reaction conditions on the Applied Biosystems 7500 Fast Real Time PCR [24]. Variants examined were: A [EX5-/KTS-], B [EX5+/KTS-], C [EX5-/KTS+] and D [EX5+/KTS+]. Analysis for the qPCR assessed relative quantification to GAPDH, in which qPCR for total WT1 and WT1 isoforms was performed over 50 cycles, in which reactions detected during cycles 11–40 were considered specific, and reactions after cycle 41 were considered unspecific [24].

### Cell block construction

Cell blocks were generated from HuARLT-1 cells, either mock vs KSHV-BAC16 infected by centrifugation of the cells, removal of supernatant and resuspension of the pellet in a mixture of fibrinogen (5mg/ml) and 5μl of thrombin (1U/μL) allowing for polymerization, followed by formalin fixation in preparation for immunohistochemistry.

### Immunohistochemistry (IHC) on KS tissue and cell blocks

Immunophenotyping was conducted on formalin-fixed, paraffin-embedded tissue sections and cell block sections on a Leica Bond III system using the standard protocol. Heat mediated antigen retrieval was used for 30 mins on the samples in pre-treatment with Sodium-Citrate buffer (pH6, epitope retrieval solution 1). The sections were then incubated with appropriate antibodies for 15 mins at room temperature and detected using an HRP-conjugated compact polymer system. 3,3’-Diaminobenzidine (DAB) was used as the chromogen. Sections were then counterstained with hematoxylin and mounted with micromount. Mouse monoclonal 6F-H2 WT1 (DAKO) and anti-LANA rat monoclonal HHV-8 ORF72 clone LN53 (Abcam) were used for IHC. T cell subsets were assessed by IHC with mouse monoclonal CD4 (clone 4B12, Leica, CD4-368-L-CE) and mouse monoclonal CD8 (clone 4B11, Leica, CD8-4B11-L-CE).

### Histopathological classification and immunohistochemistry (IHC)

All cases were reviewed by two pathologists (MH and EC) to confirm the presence of KS and were classified into one of three histologic subtypes (patch, plaque and nodule) as described [4].

### HALO analysis to quantify Immunohistochemistry staining

Immunostained slides for WT1 and LANA were scanned at 20x (Aperio, Leica Biosystems). Using HALO Imaging analysis software, WT1 and LANA staining were quantified as percent positive cells in areas involved by KS or adjacent uninvolved skin. Brown melanin staining along the epidermis was controlled for, as it was purposely excluded for WT1 analyses. WT1 was characterized based on percentage of positively stained cells, 1–20% (1+), 21–50% (2+), >50% (3+). Correlation analysis was assessed for proportions of five independent areas with high LANA or high CD8 cell numbers and the corresponding adjacent sections for other markers (LANA, CD8, CD4 and WT1). The number of cells and percentage of LANA+, CD4+ and CD8+ cells were quantified using HALO software with analysis tools, “Image Registration” and “Synchronization”. The total number of WT1+ cells, CD4+T cells, and CD8+T cells were quantified in LANA-rich areas. Conversely, five high CD8+ regions were selected and the total number of LANA+, CD8+, CD4+, and WT1+ cells were quantified in sequential sections. Proportional values of all indices were evaluated in both high LANA+ sections and high CD8+ T cells on corresponding sections with analysis performed as previously described [65]. These percentages were calculated and plotted using Graph Pad Prism version 9.4.1 for Windows (GraphPad Software, San Diego, CA, USA).

### Multiplex Immunofluorescence Tissue Staining

Multiplex Immunofluorescence Tissue Staining Multiplexed immunofluorescence (MxIF) was performed as previously described [56] using Akoya Biosciences (Marlborough, MA) by staining tissue sections from KS biopsies from the archives of Weill Cornell Medicine. The following primary antibodies were used: rat anti-LANA (Millipore LNA Anti HHV8, and Mouse monoclonal 6F-H2 WT1 (DAKO) for multiparameter imaging. A protocol of cyclical staining was utilized, with deposition of tyramide-Opal fluorophore constructs (Akoya Biosciences, Marlborough, MA) facilitated by the use of horseradish peroxidase for each cycle, with interceding application of heat, citrate-based epitope retrieval solution (Leica ER1), and Bond Wash Solution (Leica) to conduct stripping of antibody complexes between staining cycles. Finally, 4’, 6-diamidino-2-phenylindole (Spectral DAPI, Akoya Biosciences) was applied to stain nuclei. Scans of whole slides were then acquired at 20X magnification using the Vectra Polaris Automated Quantitative Pathology Imaging System (Akoya Biosciences). Scans of whole slides were tiled in Phenochart (v1.1, Akoya Biosciences); image tiles were then spectrally unmixed using InForm (v2.4.8, Akoya Biosciences). Unmixed tiles were next fused together in HALO (v3.3.2541.231, Indica Labs, Albuquerque, NM) to generate a single multilayered TIFF image file for each sample, which was then used in further analyses.

### Immunofluorescence for adherent endothelial cells

Endothelial cells were plated on collagen 22mm round #1 German glass coverslips (Corning, 354089) in 6 well plates and were then fixed by adding 2% PFA for 15 min, rocking at room temperature and then rinsed. The adherent cells on the coverslips were then permeabilized and blocked in PBS with 0.2% Saponin (Sigma-Aldrich, 84510-100G) and 5% normal goat serum (Invitrogen, 31872) for 15 min at room temperature, rocking. The coverslips were then transferred to a humidity chamber and incubated for 1 hour in PBS with 0.2% Saponin and 1% FBS with appropriate primary antibody. The coverslips were then washed, incubated with PBS and 0.2% Saponin + 1% FBS and secondary antibody for 30 min, washed and then mounted using Prolong Gold Antifade Reagent with DAPI (Cell Signaling Technology, 9071S) overnight in the dark at room temperature. Six representative images for each experimental condition were analyzed for immunofluorescence. Images were obtained using the Bio Tek Lionheart FX Automated Microscope at 20x and were exported as tiffs. The following antibodies were used: mouse monoclonal antibody WT1 clone F-6, (Santa Cruz Biotechnology, sc-7385, 1:200), and goat anti-mouse IgG conjugated to Alexa Flour 488 (Jackson ImmunoResearch, 115-545-003) was used as a secondary antibody for immunofluorescence.

### Frozen tissue sample processing for WT1 isoforms

Frozen tissue sections were acquired from Weill Cornell archives and RNA was isolated using an RNeasy Mini Kit (Qiagen, 74106) as per the standard protocol. RNA concentration was evaluated by spectrophotometry (Nanodrop, Thermo FisherScientific). The cDNA synthesis was performed using the High Capacity cDNA Reverse Transcription Kit (Thermo Fisher).

### Flow cytometry analysis

For cell surface staining, cells were incubated with the appropriate monoclonal antibodies for 30 min on ice in the dark, washed and incubated with secondary antibody reagents. Flow cytometry data were collected on a FACSCalibur (Beckton-Dickinson) and the FACS Symphony A1 (BD Biosciences) and analyzed with FlowJo V 10.7 and 10.7.1 software. The following antibodies were used for flow cytometry: a monoclonal antibody against human HLA-A2 (clone BB7.2, ThermoFisher Scientific, 17-9876-42), conjugated with APC-eFlour 780, the human monoclonal antibody ESK1 against WT1 peptide/ HLA-A0201 complex [36], and an IgG2b isotype control (BD Biosciences, 400325). For apoptosis detection, cells were stained using Annexin V (Biolegend 640920) and DAPI (Cell Signaling Technology, 4083S).

### Statistics

Statistical analyses were performed using GraphPad Prism 9.4.1 (GraphPad Software) and SAS version 9.4, with a two-sided significance level of 5%, unless otherwise stated. T-tests, analysis of variance (ANOVA), correlation analysis, and non-parametric tests (Kruskal Wallis and exact Wilcoxon tests) were used to evaluate associations.

## Disclaimer

CCG co-authored this paper in her capacity as a US Government employee but the views expressed are her own and should not be construed to represent those of the Department of State or the Department of Health and Human Services.

## Supporting information

S1 Table WT1 and LANA percent positive cells by Immunohistochemistry in KS categorized by Histopathologic Subtype. (DOCX)

S2 Table S2A–S2E Tables. WT1 expression in vivo, additional cohorts.
(DOCX)

S3 Table Characteristics of PLWH and HIV negative individuals with Kaposi sarcoma. (DOCX)

S4 Table Analysis of Immune Infiltrates and WT1/LANA in KS Nodules, Plaques and Patches. (DOCX)

S5 Table % WT1 by Response Status. (DOCX)

S6 Table RT-qPCR Primers. (DOCX)

S1 Data Excel spreadsheet containing, in separate sheets, the underlying numerical data used to generate Figs 1B, 1C, 1D, 1F, 2B, 2C, 3A-3C, 4B-4D, 4E, 4F, 5A, 5B, 5C, 6B, 6C, 6E, 7A, 7B, S1 and S2. (XLSX)

S1 Fig De novo KSHV infection in HuARLT-1 endothelial cells is latent. HuARLT-1 endothelial cells assessed by flow cytometry for mCherry (indicative of lytic reactivation) and GFP fluorescence (consistent with constitutive KSHV Infection) after de novo BAC-16 KSHV infection. At 48 hours post infection, almost no mCherry was detected in untreated KSHV infected samples compared to approximately 6% mCherry positive cells, p <0.01 (**) after treatment with 1mM sodium butyrate, and at 72 hours post infection, 1.42% in untreated vs 8% treated cells, p<0.01(**). These experiment were performed in triplicate with the aforementioned statistical analysis using unpaired, two sided student’s t-tests.
(TIF)

S2 Fig vFLIP upregulation in HuARLT-1 transduced with a doxycycline inducible pLVX vFLIP-FLAG lentivirus for wild type vFLIP vs mutant vFLIP. RT-qPCR was performed for vFLIP to confirm expression of vFLIP in the HuARLT-1 containing a doxycycline inducible pLVX vFLIP-FLAG lentivirus for wild type vFLIP vs mutant vFLIP (an NFκB-dead mutant (vFLIP^AAA(58–60)^) that renders vFLIP unable to bind IKKƴ), p<0.01(**), using unpaired, two sided student’s t-tests. No expression was seen in untransduced cells.
(TIF)

S3 Fig Inverse correlation of Immune Infiltrates and WT1/LANA in KS Nodules, Plaques and Patches. A. Representative detailed HALO analysis of both high LANA and high CD8+T cell regions, on sequential sections of the nodule from Fig 6A, showing immunohistochemistry (IHC) for CD4+T cells and WT1. B and C. Representative detailed HALO analysis of high CD8+T cell regions with corresponding sequential sections for CD4+ T cells, LANA, and WT1 positive cells for plaque and patches, representative cases. Of note, HALO software was utilized for “Image Registration” and “Synchronization”, and as the slides were not always sequential, in sections for each sample, the software detected the optimal corresponding areas, hence resulting at times in slight variability of the appearance of the corresponding rectangles, to take into account different corresponding sections.
(TIF)
