# Supplementary material for: Wilms’ tumor 1 (WT1) antigen is overexpressed in Kaposi Sarcoma and is regulated by KSHV vFLIP
Source: PLoS Pathog. 2024 Jan 8;20(1):e1011881. doi: 10.1371/journal.ppat.1011881 (PMC10898863; doi:10.1371/journal.ppat.1011881)
Supplement: S1 Table — (DOCX) [file ppat.1011881.s001.docx]

**S1 Table. WT1 and LANA percent positive cells by Immunohistochemistry (IHC) in KS categorized by Histopathologic Subtype.** Using HALO analysis software, WT1 and LANA percent positivity was determined from KS tumors from participants of the AMC066/A5263 (NCT01435018) trial, categorized by histopathologic subtype demonstrating significant LANA and WT1 percent positive cells associated with increased histopathologic subtype, p <0.001, using the Kruskal-Wallis test.

|  | | | **Histologic Subtype** | |  |  | |
| --- | --- | --- | --- | --- | --- | --- | --- |
|  | | | **Nodule**  **(N=50)** | **Plaque**  **(N=133)** | **Patch**  **(N=111)** | **(N=294)** | **P-Value** |
| % LANA+cells | | N | 49 | 124 | 94 | 267 | <.001* |
|  | | # missing | 1 | 9 | 17 | 27 |  |
|  | | Median | 35 | 11 | 6 | 11 |  |
|  | | Q1, Q3 | 18,46 | 3,18 | 1,13 | 3, 20 |  |
|  | | Min, Max | 0, 85 | 0, 79 | 0, 30 | 0, 85 |  |
|  |  | | | | | | |
| % WT1+cells | | N | 48 | 125 | 103 | 276 | <.001* |
|  | | # missing | 2 | 8 | 8 | 18 |  |
|  | | Median | 65 | 30 | 20 | 29 |  |
|  | | Q1, Q3 | 47,77 | 18,43 | 11,29 | 16, 47 |  |
|  | | Min, Max | 1, 88 | 0,81 | 2, 55 | 0,88 |  |
|  |  | | | | | | |
|  |  | | | | | | |
|  | *Kruskal-Wallis Test | | | | | | |

**.**
