# Supplementary material for: Wilms’ tumor 1 (WT1) antigen is overexpressed in Kaposi Sarcoma and is regulated by KSHV vFLIP
Source: PLoS Pathog. 2024 Jan 8;20(1):e1011881. doi: 10.1371/journal.ppat.1011881 (PMC10898863; doi:10.1371/journal.ppat.1011881)

**S2A-E Tables. WT1 expression in vivo, additional cohorts. S2A Table.** Classification of the IHC for WT1 in 276 cases from ACTG and AMC AIDS-KS trial AMC066/A5263 (NCT01435018), classified as 1+=1-20%, 2+= >20-50%, 3+=>50-100%, as determined by % positive cells by immunohistochemistry using quantitative image analysis. **S2B-E Tables.** Additional cohorts were examined for WT1 IHC, including 23 cases from Uganda, 8 cases from Stroger Hospital of Cook County, and 26 cases from New York, WCM archives, that included KS cases from PLWH and HIV negative individuals.


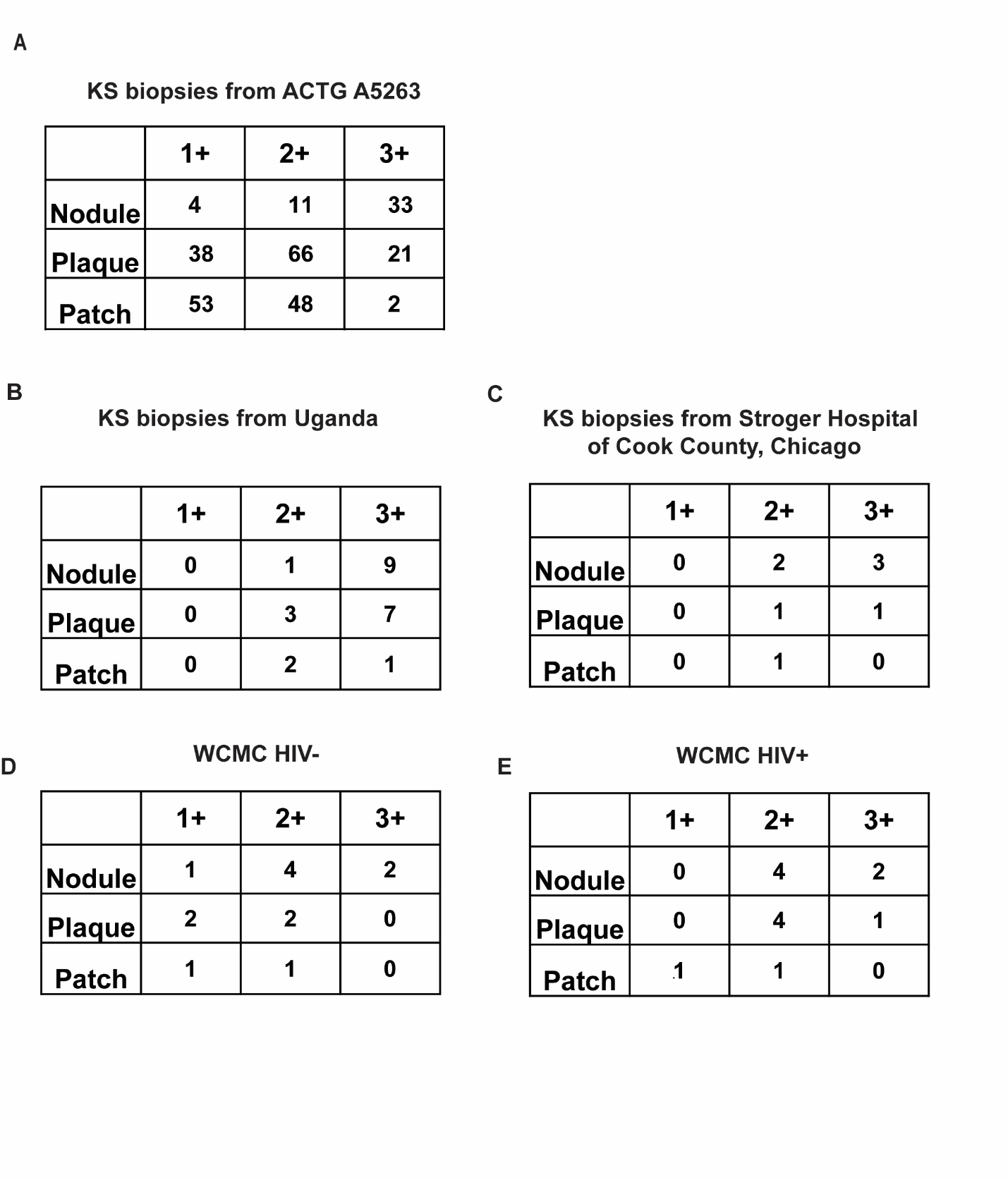

Supplement: S2 Table — WT1 expression in vivo, additional cohorts. (DOCX) [file ppat.1011881.s002.docx]
