# Supplementary material for: Wilms’ tumor 1 (WT1) antigen is overexpressed in Kaposi Sarcoma and is regulated by KSHV vFLIP
Source: PLoS Pathog. 2024 Jan 8;20(1):e1011881. doi: 10.1371/journal.ppat.1011881 (PMC10898863; doi:10.1371/journal.ppat.1011881)
Supplement: S3 Table — (DOCX) [file ppat.1011881.s003.docx]

**S3 Table. Characteristics of PLWH and HIV negative patients.** Supplemental Table S3 reports on select patient characteristics, including age, gender, race/ethnicity, and for PLWH, CD4, viral load, ART status, and the presence of extensive KS disease.

| **Characteristics of PLWH and HIV negative individuals with KS** | | | | |
| --- | --- | --- | --- | --- |
|  | **PLWH** |  | **HIV Negative** |  |
|  | **n=13** | **%** | **n=13** | **%** |
| **Age at KS presentation**** |  |  |  |  |
| <30 | 0 | 0% | 0 | 0% |
| 30-39 | 5 | 38% | 0 | 0% |
| 40-49 | 5 | 38% | 0 | 0% |
| 50-59 | 2 | 15% | 1 | 8% |
| >60 | 1 | 8% | 12 | 92% |
| **Gender** |  |  |  |  |
| Female | 2 | 15% | 2 | 15% |
| Male | 10 | 77% | 11 | 85% |
| Transgender | 1 (M to F) | 8% | 0 | 0% |
| **Race/Ethnicity** |  |  |  | 0% |
| Hispanic/Latino | 3 | 23% | 4 | 31% |
| African American | 6 | 46% | 0 | 0% |
| White | 0 | 0% | 4 | 31% |
| Other/Unknown | 4 | 31% | 5 | 46% |
| **CD4 cells/μL** |  |  |  |  |
| <200 | 8 | 62% |  |  |
| 200-349 | 1 | 8% |  |  |
| 350-499 | 2 | 15% |  |  |
| >500 | 1 | 8% |  |  |
| Unknown | 1 | 8% |  |  |
| **HIV RNA** |  |  |  |  |
| 0-499 | 3 | 23% |  |  |
| 500-4999 | 1 | 8% |  |  |
| 5000-49,999 | 2 | 15% |  |  |
| 50,000-499,999 | 4 | 31% |  |  |
| 500,000+ | 2 | 15% |  |  |
| Unknown | 1 | 0% |  |  |
| **ART** |  |  |  |  |
| Yes | 3 | 23% |  |  |
| No | 4 | 31% |  |  |
| Non-adherent | 5 | 38% |  |  |
| Unknown | 1 | 8% |  |  |
| **Visceral/advanced disease** |  |  |  |  |
| Yes | 3 | 23% | 0 | 0% |
| No | 9 | 69% | 12 | 92% |
| Unknown | 1 | 8% | 1 | 8% |
| **This documented presentation may be a new recurrent episode of KS and not necessarily the initial diagnostic biopsy. | | | | |
