# Supplementary material for: Wilms’ tumor 1 (WT1) antigen is overexpressed in Kaposi Sarcoma and is regulated by KSHV vFLIP
Source: PLoS Pathog. 2024 Jan 8;20(1):e1011881. doi: 10.1371/journal.ppat.1011881 (PMC10898863; doi:10.1371/journal.ppat.1011881)
Supplement: S4 Table — (DOCX) [file ppat.1011881.s004.docx]

**S4Table. Analysis of Immune Infiltrates and WT1/LANA in KS Nodules, Plaques and Patches.** Analysis performed using one way ANOVA, Tukey’s multiple comparison test for high LANA regions in nodules, and high CD8+ regions in peripheral stroma of nodules, and in plaques and patches.

| **Ordinary one-way ANOVA of Nodule High LANA Regions**   \| Tukey's multiple comparisons test \| Mean Diff. \| 95.00% CI of diff. \| Below threshold \| Summary \| Adjusted P Value \|  \| \| --- \| --- \| --- \| --- \| --- \| --- \| --- \| \| LANA vs. WT1 \| -30.86 \| -67.79 to 6.069 \| No \| ns \| 0.1289 \| A-B \| \| LANA vs. CD8 \| 43.37 \| 20.76 to 65.99 \| Yes \| **** \| <0.0001 \| A-C \| \| LANA vs. CD4 \| 42.98 \| 18.72 to 67.23 \| Yes \| *** \| 0.0002 \| A-D \| \| WT1 vs. CD8 \| 74.23 \| 37.30 to 111.2 \| Yes \| **** \| <0.0001 \| B-C \| \| WT1 vs. CD4 \| 73.84 \| 35.88 to 111.8 \| Yes \| **** \| <0.0001 \| B-D \| \| CD8 vs. CD4 \| -0.3980 \| -24.65 to 23.85 \| No \| ns \| >0.9999 \| C-D \| \|  \|  \|  \|  \|  \|  \|  \| \| Test details \| Mean 1 \| Mean 2 \| Mean Diff. \| SE of diff. \| n1 \| n2 \| \| LANA vs. WT1 \| 59.76 \| 90.62 \| -30.86 \| 13.69 \| 13 \| 3 \| \| LANA vs. CD8 \| 59.76 \| 16.39 \| 43.37 \| 8.385 \| 13 \| 13 \| \| LANA vs. CD4 \| 59.76 \| 16.78 \| 42.98 \| 8.992 \| 13 \| 10 \| \| WT1 vs. CD8 \| 90.62 \| 16.39 \| 74.23 \| 13.69 \| 3 \| 13 \| \| WT1 vs. CD4 \| 90.62 \| 16.78 \| 73.84 \| 14.07 \| 3 \| 10 \| \| CD8 vs. CD4 \| 16.39 \| 16.78 \| -0.3980 \| 8.992 \| 13 \| 10 \| \| \| **Ordinary one-way ANOVA of Nodule High CD8 Regions** \| \| \| \| \| \| \| \| --- \| --- \| --- \| --- \| --- \| --- \| --- \| \| Tukey's multiple comparisons test \| Mean Diff. \| 95.00% CI of diff. \| Below threshold \| Summary \| Adjusted P Value \|  \| \| CD8 vs. CD4 \| 42.34 \| 26.79 to 57.90 \| Yes \| **** \| <0.0001 \| A-B \| \| CD8 vs. LANA \| 63.79 \| 49.29 to 78.30 \| Yes \| **** \| <0.0001 \| A-C \| \| CD8 vs. WT1 \| 54.31 \| 30.62 to 78.00 \| Yes \| **** \| <0.0001 \| A-D \| \| CD4 vs. LANA \| 21.45 \| 5.892 to 37.01 \| Yes \| ** \| 0.0037 \| B-C \| \| CD4 vs. WT1 \| 11.96 \| -12.38 to 36.31 \| No \| ns \| 0.5535 \| B-D \| \| LANA vs. WT1 \| -9.485 \| -33.17 to 14.21 \| No \| ns \| 0.7040 \| C-D \| \|  \|  \|  \|  \|  \|  \|  \| \| Test details \| Mean 1 \| Mean 2 \| Mean Diff. \| SE of diff. \| n1 \| n2 \| \| CD8 vs. CD4 \| 67.42 \| 25.07 \| 42.34 \| 5.768 \| 13 \| 10 \| \| CD8 vs. LANA \| 67.42 \| 3.626 \| 63.79 \| 5.379 \| 13 \| 13 \| \| CD8 vs. WT1 \| 67.42 \| 13.11 \| 54.31 \| 8.784 \| 13 \| 3 \| \| CD4 vs. LANA \| 25.07 \| 3.626 \| 21.45 \| 5.768 \| 10 \| 13 \| \| CD4 vs. WT1 \| 25.07 \| 13.11 \| 11.96 \| 9.028 \| 10 \| 3 \| \| LANA vs. WT1 \| 3.626 \| 13.11 \| -9.485 \| 8.784 \| 13 \| 3 \| \| \| \| \| \| \| \| \| **Ordinary one-way ANOVA of Plaque High CD8 Regions** \| \| \| \| \| \| \| |
| --- | --- | --- | --- | --- | --- | --- | --- | --- | --- | --- | --- | --- | --- | --- | --- | --- | --- | --- | --- | --- | --- | --- | --- | --- | --- | --- | --- | --- | --- | --- | --- | --- | --- | --- | --- | --- | --- | --- | --- | --- | --- | --- | --- | --- | --- | --- | --- | --- | --- | --- | --- | --- | --- | --- | --- | --- | --- | --- | --- | --- | --- | --- | --- | --- | --- | --- | --- | --- | --- | --- | --- | --- | --- | --- | --- | --- | --- | --- | --- | --- | --- | --- | --- | --- | --- | --- | --- | --- | --- | --- | --- | --- | --- | --- | --- | --- | --- | --- | --- | --- | --- | --- | --- | --- | --- | --- | --- | --- | --- | --- | --- | --- | --- | --- | --- | --- | --- | --- | --- | --- | --- | --- | --- | --- | --- | --- | --- | --- | --- | --- | --- | --- | --- | --- | --- | --- | --- | --- | --- | --- | --- | --- | --- | --- | --- | --- | --- | --- | --- | --- | --- | --- | --- | --- | --- | --- | --- | --- | --- | --- | --- | --- | --- | --- | --- | --- | --- | --- | --- | --- | --- | --- | --- | --- | --- | --- | --- | --- | --- | --- | --- | --- | --- | --- | --- | --- | --- | --- | --- | --- | --- | --- | --- | --- | --- | --- | --- | --- | --- | --- | --- | --- | --- | --- | --- | --- | --- | --- | --- | --- | --- | --- | --- | --- | --- | --- | --- | --- | --- | --- | --- | --- | --- | --- | --- | --- | --- | --- | --- | --- | --- |
| \| Tukey's multiple comparisons test \| Mean Diff. \| 95.00% CI of diff. \| Below threshold \| Summary \| Adjusted P Value \|  \| \| --- \| --- \| --- \| --- \| --- \| --- \| --- \| \| CD8 vs. CD4 \| 30.66 \| 11.90 to 49.42 \| Yes \| *** \| 0.0005 \| A-B \| \| CD8 vs. LANA \| 54.42 \| 36.11 to 72.73 \| Yes \| **** \| <0.0001 \| A-C \| \| CD8 vs. WT1 \| 37.09 \| 15.30 to 58.88 \| Yes \| *** \| 0.0003 \| A-D \| \| CD4 vs. LANA \| 23.76 \| 5.000 to 42.52 \| Yes \| ** \| 0.0085 \| B-C \| \| CD4 vs. WT1 \| 6.431 \| -15.74 to 28.60 \| No \| ns \| 0.8614 \| B-D \| \| LANA vs. WT1 \| -17.33 \| -39.12 to 4.462 \| No \| ns \| 0.1588 \| C-D \| \|  \|  \|  \|  \|  \|  \|  \| \| Test details \| Mean 1 \| Mean 2 \| Mean Diff. \| SE of diff. \| n1 \| n2 \| \| CD8 vs. CD4 \| 59.01 \| 28.35 \| 30.66 \| 6.946 \| 11 \| 10 \| \| CD8 vs. LANA \| 59.01 \| 4.588 \| 54.42 \| 6.779 \| 11 \| 11 \| \| CD8 vs. WT1 \| 59.01 \| 21.92 \| 37.09 \| 8.068 \| 11 \| 6 \| \| CD4 vs. LANA \| 28.35 \| 4.588 \| 23.76 \| 6.946 \| 10 \| 11 \| \| CD4 vs. WT1 \| 28.35 \| 21.92 \| 6.431 \| 8.209 \| 10 \| 6 \| \| LANA vs. WT1 \| 4.588 \| 21.92 \| -17.33 \| 8.068 \| 11 \| 6 \| \|  \| \| \| \| \| \| \| |
| **Ordinary one-way ANOVA of Patch High CD8 Regions** |
| \| Tukey's multiple comparisons test \| Mean Diff. \| 95.00% CI of diff. \| Below threshold \| Summary \| Adjusted P Value \|  \| \| --- \| --- \| --- \| --- \| --- \| --- \| --- \| \| CD8 vs. CD4 \| 32.84 \| 13.79 to 51.90 \| Yes \| *** \| 0.0003 \| A-B \| \| CD8 vs. LANA \| 60.67 \| 46.05 to 75.28 \| Yes \| **** \| <0.0001 \| A-C \| \| CD8 vs. WT1 \| 48.35 \| 32.57 to 64.14 \| Yes \| **** \| <0.0001 \| A-D \| \| CD4 vs. LANA \| 27.82 \| 8.767 to 46.88 \| Yes \| ** \| 0.0021 \| B-C \| \| CD4 vs. WT1 \| 15.51 \| -4.457 to 35.48 \| No \| ns \| 0.1742 \| B-D \| \| LANA vs. WT1 \| -12.31 \| -28.10 to 3.474 \| No \| ns \| 0.1715 \| C-D \| \|  \|  \|  \|  \|  \|  \|  \| \| Test details \| Mean 1 \| Mean 2 \| Mean Diff. \| SE of diff. \| n1 \| n2 \| \| CD8 vs. CD4 \| 65.93 \| 33.09 \| 32.84 \| 7.056 \| 12 \| 5 \| \| CD8 vs. LANA \| 65.93 \| 5.263 \| 60.67 \| 5.412 \| 12 \| 12 \| \| CD8 vs. WT1 \| 65.93 \| 17.58 \| 48.35 \| 5.845 \| 12 \| 9 \| \| CD4 vs. LANA \| 33.09 \| 5.263 \| 27.82 \| 7.056 \| 5 \| 12 \| \| CD4 vs. WT1 \| 33.09 \| 17.58 \| 15.51 \| 7.394 \| 5 \| 9 \| \| LANA vs. WT1 \| 5.263 \| 17.58 \| -12.31 \| 5.845 \| 12 \| 9 \| |
