# Supplementary material for: Wilms’ tumor 1 (WT1) antigen is overexpressed in Kaposi Sarcoma and is regulated by KSHV vFLIP
Source: PLoS Pathog. 2024 Jan 8;20(1):e1011881. doi: 10.1371/journal.ppat.1011881 (PMC10898863; doi:10.1371/journal.ppat.1011881)
Supplement: S5 Table — (DOCX) [file ppat.1011881.s005.docx]

**S5 Table: % WT1 by Response Status.** The percent positivity of WT1 expression of KS tumors by immunohistochemistry determined using HALO analysis software of participants, all with advanced stage KS from the AMC066/A5263 (NCT01435018) trial did not associate with whether a participate was considered a ‘good responder’ or whether they were considered a ‘progressor’.

|  | | **Response Status** | |  | |
| --- | --- | --- | --- | --- | --- |
|  | | **Good Responder**  **(N=69)** | **Progressor**  **(N=68)** | **Total**  **(N=137)** | **P-Value** |
| % WT1+ cells | N | 66 | 65 | 131 | 0.519* |
|  | # missing | 3 | 3 | 6 |  |
|  | Median | 24.37 | 29.32 | 27.68 |  |
|  | Q1, Q3 | 17.68, 40.45 | 14.26, 50.29 | 15.93, 43.87 |  |
|  | Min, Max | 0.35, 83.90 | 2.06, 88.06 | 0.35, 88.06 |  |
|  | | | | | |
| *Exact Wilcoxon Test | | | | | |
