# Supplementary material for: Wilms’ tumor 1 (WT1) antigen is overexpressed in Kaposi Sarcoma and is regulated by KSHV vFLIP
Source: PLoS Pathog. 2024 Jan 8;20(1):e1011881. doi: 10.1371/journal.ppat.1011881 (PMC10898863; doi:10.1371/journal.ppat.1011881)
Supplement: S6 Table — (DOCX) [file ppat.1011881.s006.docx]

**S6 Table. RT-qPCR Primers**

| **RT-qPCR primers** |  |
| --- | --- |
| WT1-forward | 5'- ACA GGG TAC GAG AGC GAT AA-3' |
| WT1-reverse | 5'- CAC ATC CTG AAT GCC TCT GAA-3' |
| vFLIP-forward | 5'- CCA TAC AGT ACA CCC AGT GTA AG-3' |
| vFLIP-reverse | 5'- GCT GTG TGC GAG GGA TATT-3' |
| LANA-forward | 5'-GCC TAT ACC AGG AAG TCC CA-3' |
| LANA-reverse | 5'-GAG CCA CCG GTA AAG TAG GA -3' |
| K8.1-forward | 5' AAT ATC AGC CTT TTC AGG ATC A- 3' |
| K8.1-reverse | 5'CAC CAC TAT TTC TGC CGT TTT C3' |
| Bcl-2-forward | 5' CAT GCT GGG GCC GTA CAG- 3' |
| Bcl-2-reverse | 5' GAA CCG GCA CCT GCA CAC- 3' |
| B-actin forward | 5'- CCC AGC ACA ATG AAG ATC AAG ATC AT-3' |
| B-actin reverse | 5'- ATC TGC TGG AAG GTG GAC AGC GA-3' |
| GAPDH- forward | 5'- ACT GCC ACC CAG AAG ACT GT-3' |
| GAPDH- reverse | 5'- CCA GTA GAG GCA GGG ATG AT-3' |
| WT1-total-forward | 5'- TAC ACA CGC ACG GTG TCT TCA-3' |
| WT1-total-reverse | 5'- CTC AGA TGC CGA CCA TAC AAG-3' |
| EX5(-) forward | 5'- GAG CCA CCT TAA AGG GCC A-3' |
| EX5(+) forward | 5'- ATG GAC AGA AGG GCA GAG CA-3' |
| KTS(-) reverse | 5'- GCT GAA GGG CTT TTC ACC TGT A-3' |
| KTS(+) reverse | 5'- CTG AAG GGC TTT TCA CTT GTT TTA C-3' |
|  |  |
| **Combinations** |  |
| WT1—total | WT1-total-forward ---- WT1-total-reverse |
| WT1—isoform A | EX5(-) forward ---- KTS(-) reverse |
| WT1—isoform B | EX5(+) forward ---- KTS(-) reverse |
| WT1—isoform C | EX5(-) forward ---- KTS(+) reverse |
| WT1—isoform D | EX5(+) forward---- KTS(+) reverse |
