# Supplementary figures and images for: Wilms’ tumor 1 (WT1) antigen is overexpressed in Kaposi Sarcoma and is regulated by KSHV vFLIP
Source: PLoS Pathog. 2024 Jan 8;20(1):e1011881. doi: 10.1371/journal.ppat.1011881 (PMC10898863; doi:10.1371/journal.ppat.1011881)

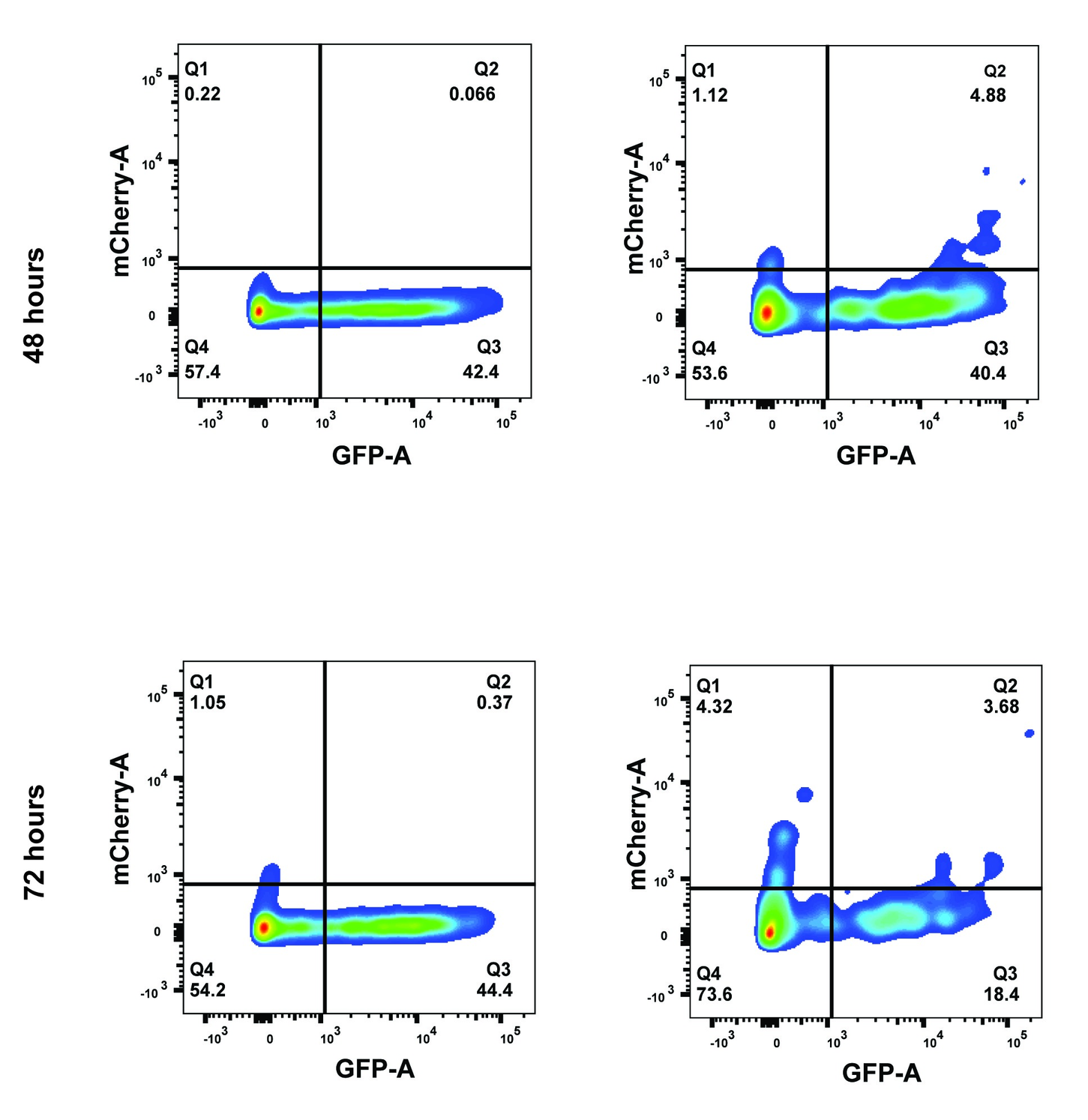

Supplement: S1 Fig — HuARLT-1 endothelial cells assessed by flow cytometry for mCherry (indicative of lytic reactivation) and GFP fluorescence (consistent with constitutive KSHV Infection) after de novo BAC-16 KSHV infection. At 48 hours post infection, almost no mCherry was detected in untreated KSHV infected samples compared to approximately 6% mCherry positive cells, p <0.01 (**) after treatment with 1mM sodium butyrate, and at 72 hours post infection, 1.42% in untreated vs 8% treated cells, p<0.01(**). These experiment were performed in triplicate with the aforementioned statistical analysis using unpaired, two sided student’s t-tests. (TIF) [file ppat.1011881.s008.tif]

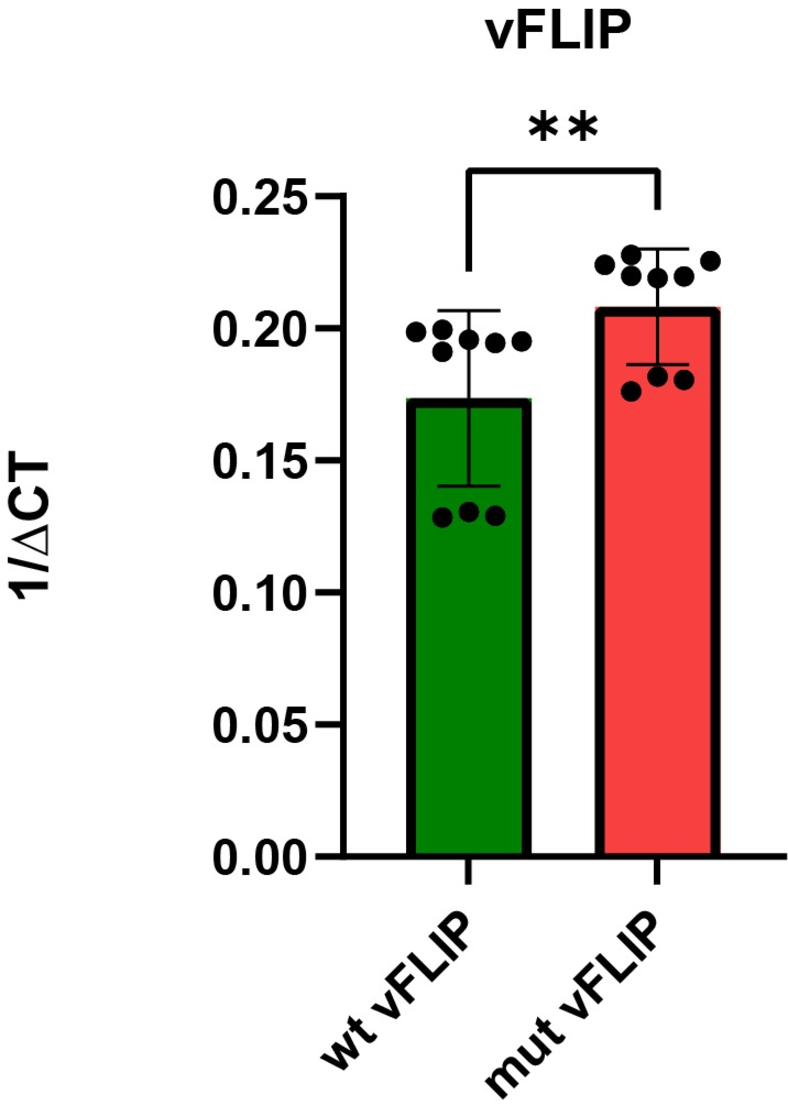

Supplement: S2 Fig — RT-qPCR was performed for vFLIP to confirm expression of vFLIP in the HuARLT-1 containing a doxycycline inducible pLVX vFLIP-FLAG lentivirus for wild type vFLIP vs mutant vFLIP (an NFκB-dead mutant (vFLIPAAA(58–60)) that renders vFLIP unable to bind IKKƴ), p<0.01(**), using unpaired, two sided student’s t-tests. No expression was seen in untransduced cells. (TIF) [file ppat.1011881.s009.tif]

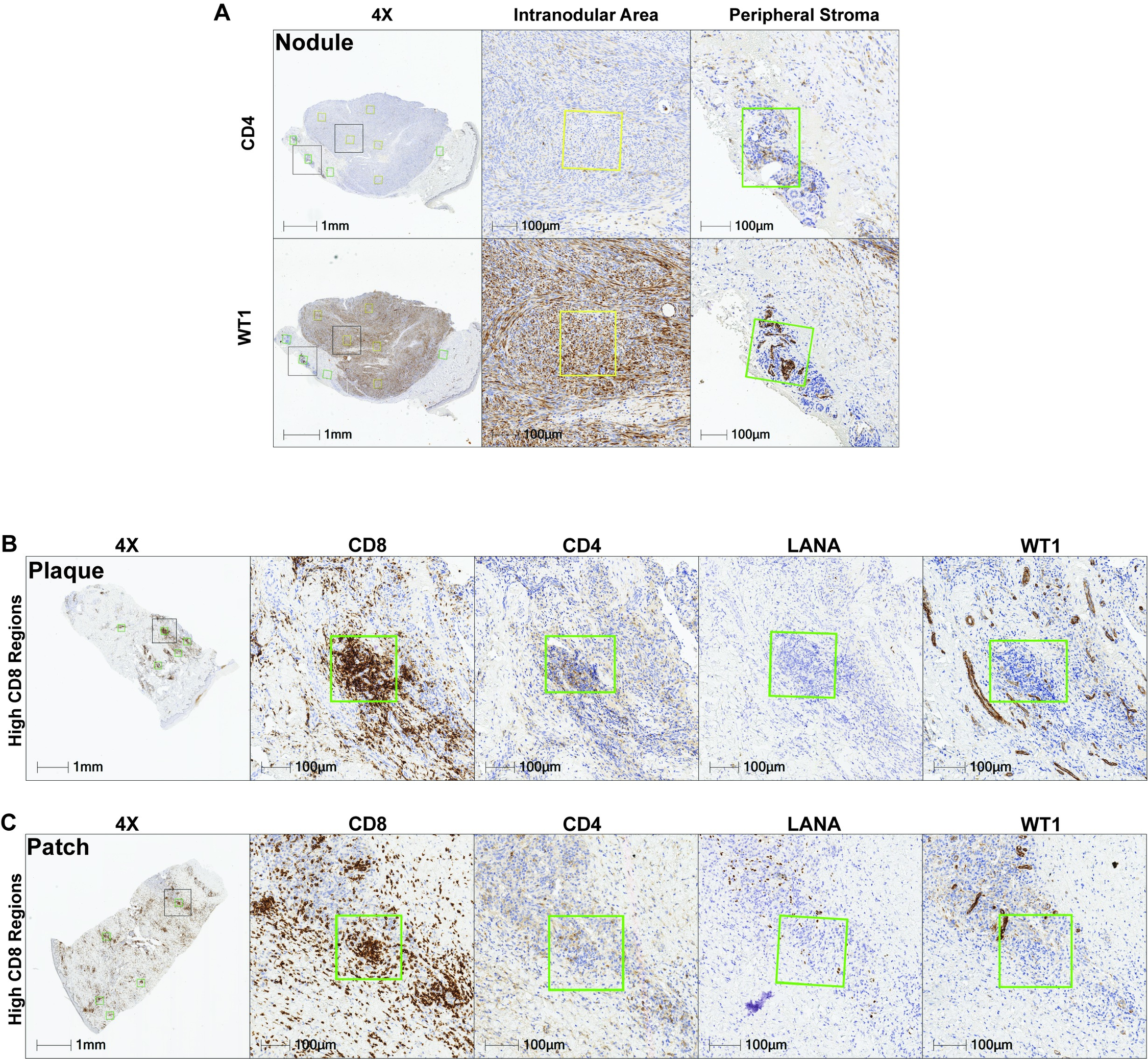

Supplement: S3 Fig — A. Representative detailed HALO analysis of both high LANA and high CD8+T cell regions, on sequential sections of the nodule from Fig 6A, showing immunohistochemistry (IHC) for CD4+T cells and WT1. B and C. Representative detailed HALO analysis of high CD8+T cell regions with corresponding sequential sections for CD4+ T cells, LANA, and WT1 positive cells for plaque and patches, representative cases. Of note, HALO software was utilized for “Image Registration” and “Synchronization”, and as the slides were not always sequential, in sections for each sample, the software detected the optimal corresponding areas, hence resulting at times in slight variability of the appearance of the corresponding rectangles, to take into account different corresponding sections. (TIF) [file ppat.1011881.s010.tif]
